# Telehealth System Based on the Ontology Design of a Diabetes Management Pathway Model in China: Development and Usability Study

**DOI:** 10.2196/42664

**Published:** 2022-12-19

**Authors:** ZhiYuan Fan, LiYuan Cui, Ying Ye, ShouCheng Li, Ning Deng

**Affiliations:** 1 College of Biomedical Engineering and Instrument Science Ministry of Education Key Laboratory of Biomedical Engineering Zhejiang University Hangzhou China; 2 Binjiang Institute of Zhejiang University Hangzhou China; 3 School of Medical Imaging Hangzhou Medical College HangZhou China

**Keywords:** diabetes, chronic disease management, Chronic Disease Management Pathway, ontology, Semantic Web Rule Language rules, SWRL rules

## Abstract

**Background:**

Diabetes needs to be under control through management and intervention. Management of diabetes through mobile health is a practical approach; however, most diabetes mobile health management systems do not meet expectations, which may be because of the lack of standardized management processes in the systems and the lack of intervention implementation recommendations in the management knowledge base.

**Objective:**

In this study, we aimed to construct a diabetes management care pathway suitable for the actual situation in China to express the diabetes management care pathway using ontology and develop a diabetes closed-loop system based on the construction results of the diabetes management pathway and apply it practically.

**Methods:**

This study proposes a diabetes management care pathway model in which the management process of diabetes is divided into 9 management tasks, and the Diabetes Care Pathway Ontology (DCPO) is constructed to represent the knowledge contained in this pathway model. A telehealth system, which can support the comprehensive management of patients with diabetes while providing active intervention by physicians, was designed and developed based on the DCPO. A retrospective study was performed based on the data records extracted from the system to analyze the usability and treatment effects of the DCPO.

**Results:**

The diabetes management pathway ontology constructed in this study contains 119 newly added classes, 28 object properties, 58 data properties, 81 individuals, 426 axioms, and 192 Semantic Web Rule Language rules. The developed mobile medical system was applied to 272 patients with diabetes. Within 3 months, the average fasting blood glucose of the patients decreased by 1.34 mmol/L (*P*=.003), and the average 2-hour postprandial blood glucose decreased by 2.63 mmol/L (*P*=.003); the average systolic and diastolic blood pressures decreased by 11.84 mmHg (*P*=.02) and 8.8 mmHg (*P*=.02), respectively. In patients who received physician interventions owing to abnormal attention or low-compliance warnings, the average fasting blood glucose decreased by 2.45 mmol/L (*P*=.003), and the average 2-hour postprandial blood glucose decreased by 2.89 mmol/L (*P*=.003) in all patients with diabetes; the average systolic and diastolic blood pressure decreased by 20.06 mmHg (*P*=.02) and 17.37 mmHg (*P*=.02), respectively, in patients with both hypertension and diabetes during the 3-month management period.

**Conclusions:**

This study helps guide the timing and content of interactive interventions between physicians and patients and regulates physicians’ medical service behavior. Different management plans are formulated for physicians and patients according to different characteristics to comprehensively manage various cardiovascular risk factors. The application of the DCPO in the diabetes management system can provide effective and adequate management support for patients with diabetes and those with both diabetes and hypertension.

## Introduction

### Background

Diabetes mellitus is one of the most common chronic diseases in China [1]. People with diabetes have an increased risk of developing serious health problems [2]. In patients with diabetes, consistent high blood glucose (BG) levels can lead to serious diseases affecting the heart and blood vessels [3]. The combination of lifestyle modifications and self-care therapies as part of diabetes management can significantly increase the treatment rate of diabetes, reduce the incidence of cardiovascular disease, and improve the quality of life of patients [4]. The chronic care model is the most widely used chronic disease management model, which emphasizes that physicians and patients participate in the management process together for collaborative management [5,6]. The purpose of the chronic care model is to remind physicians to provide patients with timely and efficient management, which means the physician will immediately intervene and give feedback on the patient’s behavior when the patients complete the management tasks [7]. However, the traditional diabetes management method cannot meet the long-term management needs of patients owing to time and space constraints [8]. In addition, most patients with diabetes have multiple cardiovascular risk factors, such as hypertension, hyperglycemia, and obesity, which are the main causes of death in patients with diabetes [9]. Therefore, in addition to the comprehensive management of patients with diabetes based on the BG level, interventions to control multiple cardiovascular risk factors are also required. With the development of mobile internet technology, diabetes management tends to be digitalized, which relieves the time and space limitations of traditional management methods and realizes the dynamic monitoring and maintenance of patients throughout the entire process managed by medical service providers [10].

### Previous Work

Compared with traditional diabetes management methods, the application of mobile medical technology can change the role of patients from passively accepting management services to having the core role in management work, positively improving their self-management awareness. Wyne et al [11] used mobile health technology to manage patients with type 2 diabetes mellitus (T2DM) and effectively prevented serious complications, demonstrating that mobile health technology improved the management of patients with diabetes. Quinn et al [12] conducted a cluster-randomized trial using the BlueStar diabetes care system (WellDoc Inc). The trial results showed that the glycosylated hemoglobin level was significantly reduced and the depression levels and other physiological indicators (blood pressure [BP], lipids, etc) were also improved in the patients engaged in the care and intervention of this system over a 1-year treatment period.

It is necessary to transform medical knowledge and clinical data into computer-recognizable knowledge models using the Semantic Web. As a formal representation of knowledge that can accurately describe the relationship between concepts, ontology has gradually become a key technology for realizing the Semantic Web. The use of ontology to express domain knowledge can facilitate knowledge sharing and dissemination. It is also key to realizing a complete knowledge base and an intelligent clinical decision support system. El-Sappagh et al [13] constructed the Diabetes Diagnosis Ontology based on diabetes clinical practice guidelines and principles of standard medical terminology, established Semantic Web Rule Language (SWRL) diagnostic rules, and used an inference engine to perform diagnostic inference on the T2DM diagnostic knowledge base. Krishnan et al [14] constructed the Diabetes Mellitus Treatment Ontology as a basis for sharing semantic, domain-specific, standard, machine-readable, and interoperable knowledge related to T2DM treatment. Fast Healthcare Interoperability Resources and Semantic Sensor Network–based type 1 diabetes mellitus Ontology is designed for managing patients with type 1 diabetes, which has the Health Level 7-Fast Healthcare Interoperability Resources standard and Semantic Sensor Network sensor ontology integrated, and provides patients with a complete and personalized treatment plan based on the complete patient information [15]. Sherimon et al [16] proposed an ontology-based clinical decision support system for patients with diabetes, which predicts the risk of patients according to various risk factors, including smoking, alcohol consumption, and cardiovascular family history. Chen et al [17] proposed an ontology-based model for the diagnosis and treatment of patients with diabetes who are in hospital, and it can help reduce medication errors.

### Key Issues

Previous studies have used diabetes clinical practice guidelines as the theoretical basis for the use of ontologies and considered patient profile data in knowledge-based systems for decision support in hospitals and in-home diabetes monitoring and management, including patient self-monitoring, data recording, and physicians’ simple management advice. However, because of the lack of clear regulations on physician-patient interaction and intervention feedback mechanisms in medical guidelines, these studies ignore the importance of physicians’ active intervention on patients, do not define the interactive intervention mechanism between physicians and patients, and lack active intervention time and initiative [15-17]. The definition of intervention content and digital management recommendations failed to combine standardized processes and evidence-based medical knowledge, resulting in the inability of patients with diabetes to receive effective intervention guidance promptly in actual management. No research has considered the appropriate timing and details of physician intervention in patients with diabetes when constructing diabetes management ontology.

Furthermore, the existing ontology construction process focuses on hyperglycemia control in patients with diabetes, ignoring the importance of controlling and managing other cardiovascular risk factors and treating these factors as patient-related indicators without targeted attention and guidance in the care process.

### Objective

A previous study proposed the concept and construction method for the Chronic Disease Management Pathway (CDMP) [18,19]. CDMP has been proven effective in the treatment of hypertension and chronic obstructive pulmonary disease [20,21]. It is becoming one of the practical approaches for chronic disease management owing to its continuous, closed-loop, and standardized features and provides reliable implementation guidelines for chronic disease management. The introduction and application of the CDMP offer a new solution for the digital management of patients with diabetes.

In this study, we extracted the key issues in diabetes management through evidence-based medical guidelines and expert recommendations, combined the issues with the CDMP approach, and constructed the Type 2 Diabetes Mellitus Management Pathway (T2DMMP) model. A closed-loop Type 2 Diabetes Mellitus Management System (T2DMMS) was developed based on the T2DMMP model, which was expressed by ontology modeling to ensure that computer operations could execute it. Our management pathway was shown to be usable and effective in clinical diabetes management as an implementable intervention mechanism for physician-patient interactions.

## Methods

### Type 2 Diabetes Mellitus Management Pathway Model

#### Overview

The diagnosis and management of diabetes mellitus is a complex process. To help with the diabetes management process, in this study, the T2DMMP was constructed based on numerous diabetes prevention guidelines and other cardiovascular risk factors, focusing on clarifying the responsibilities of management roles and providing a standardized and complete management pathway for comprehensive diabetes management. The T2DMMP is a process that divides management tasks into physician intervention plans and patient self-management plans by defining 2 different management roles for physicians and patients.

#### Summary Extraction of Task Sets

The following literature on current diabetes prevention and management guidelines with high recognition was collected through literature research, and the task sets were extracted from them: Guidelines for the prevention and control of type 2 diabetes in China (2017) Edition [22], National guidelines for the prevention and control of diabetes in primary care (2018) [23], the comprehensive type 2 diabetes management algorithm [24], Management of Hyperglycemia in Type 2 Diabetes [25], and Diabetes Canada Clinical Practice Guidelines Expert Committee [26]. The analysis of the core content of diabetes management was performed based on the guidelines for the prevention and control of type 2 diabetes in China (2017 Edition) and National guidelines for the prevention and control of diabetes in primary care (2018).

#### Type 2 Diabetes Mellitus Management Pathway

**Figure 1:**
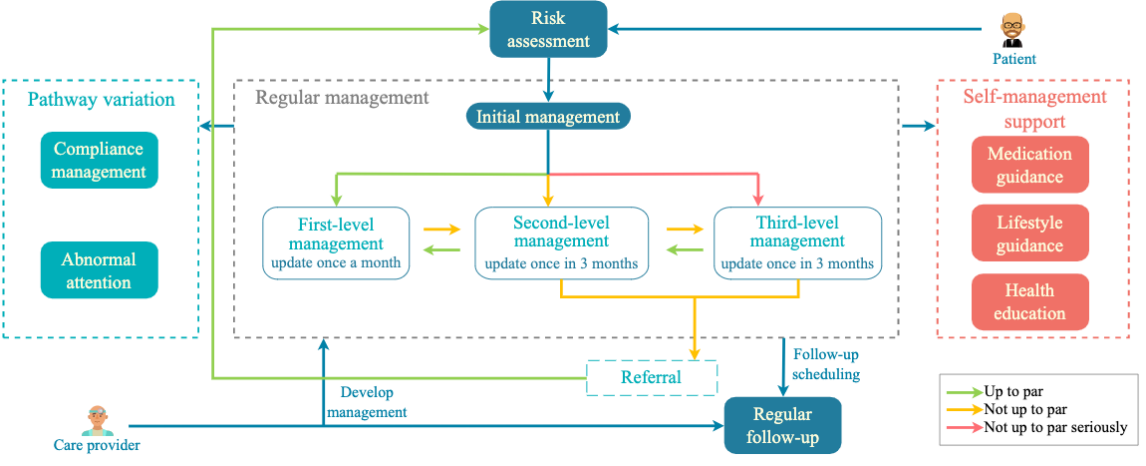
Diagram of the type 2 diabetes mellitus management pathway.

With regard to the CDMP model, 9 common tasks were defined in the T2DMMP, which could be grouped into 3 parts as follows: the task set for regular management, task set for pathway variation, and task set for self-management support. A pathway map of T2DMMP is shown in Figure 1. When a patient is diagnosed with diabetes, they will enter the diabetes management care pathway. A comprehensive cardiovascular risk assessment will be conducted subsequently, and different management levels will be formulated according to the assessment results as follows: patients with stable blood sugar are at routine first-level management; patients with unsatisfactory blood sugar are at routine second-level management; and patients with fasting BG (FBG) >11.1 mmol/L are in intensive third-level management. Different management plans are provided to patients at different management levels. The patient’s condition will be periodically evaluated according to the patient’s self-monitoring data, and the patient will be dynamically adjusted to the appropriate management level in the management path.

The task set for pathway variation consists of 2 tasks as follows: abnormal attention and compliance management. Once the patient’s self-monitoring data (such as BG or BP) is abnormal, the caregiver needs to intervene immediately and appropriately. Once the patient’s compliance is low, the care provider needs to conduct additional follow-up to motivate the patient.

The task set for self-management support consists of the following 3 tasks: medication guidance, lifestyle guidance, and health education. Medication guidance is designed to provide medication treatment plans, whereas lifestyle guidance provides non–drug treatment plans such as diet and physical activity. Health education aims to increase patients’ awareness of the disease, thereby improving their self-management skills.

Two parties will play a role in management: the care provider and the patient. The care provider team comprises general practitioners, case managers in community health services, and specialists in secondary or tertiary hospitals. According to this pathway, care providers should work collaboratively for day-to-day management, such as regular follow-up and interventions for abnormal conditions. Patients need self-monitoring according to their self-management plans and receive timely intervention from their care providers.

### Construction of Diabetes Care Pathway Ontology

#### Overview

**Figure 2:**
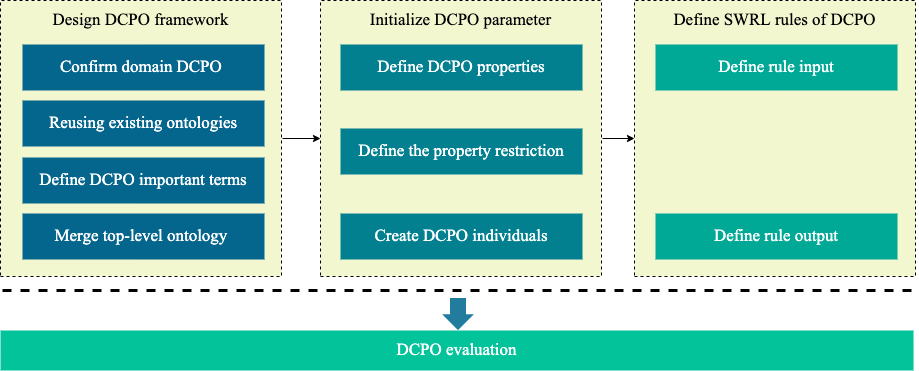
The Diabetes Care Pathway Ontology (DCPO) construction methodology. SWRL: Semantic Web Rule Language.

Ontology represents domain knowledge in a machine-readable and formal format [27]. It can be incorporated into a clinical decision support system as a knowledge base [28,29]. The Diabetes Care Pathway Ontology (DCPO) was constructed to describe the concept of T2DMMP. As shown in Figure 2, the DCPO is divided into 3 phases. Stages 1 and 2 follow the ontology engineering approach widely used to represent the structural information of the model, whereas stage 3 incorporates the medical knowledge of the model through external semantic rules.

#### Stage 1: DCPO Framework Design

At this stage, a set of questions that the ontology should answer the domain and scope of the DCPO are listed—the so-called capability questions. In this step, the DCPO will be identified as a representative of the T2DMMP model. The expected output of the DCPO is the monitoring and management of patients with diabetes, which includes individualized treatment plans and specific management tasks for physicians and patients.

On the basis of the scalability, standardization, and reusability of the ontologies, already existing diabetes ontologies were considered reusable. Keywords such as “diabetes management,” “diabetes ontology,” “diabetes medication ontology,” “diabetes diet ontology,” and “diabetes treatment ontology” were used to search for content on the BioPortal and PubMed websites. Diabetes Diagnosis Ontology and Diabetes Mellitus Treatment Ontology were also identified for use. To achieve the diabetes management path, the *TIME.owl* ontology of the W3C (World Wide Web Consortium) standard was used as the temporality module. It defines the timing of the management tasks by defining the time intervals and moments. Broad coverage of diabetes treatment medication drugs was introduced, and the drug-drug interaction ontology was reused to describe the types of drugs in the medication guidance module. The lifestyle module included a diet plan and an exercise plan. The diet plan was a set of dietary recommendations for the patient; therefore, the *OntoFood* ontology was reused. On the basis of the T2DMMP model, the DCPO model is defined as a class and class hierarchy and is divided into the following 2 primary levels of abstraction: the first level for the core concepts in the path and the second level for the detailed elements of the first-level classes. The first-level core concepts include patient profile, management roles, management plans, management tasks, and chronological expressions. The patient’s self-management plan and the physician’s diagnosis and treatment plan defined under the management plan are the contents of the second level. This definition can accurately describe the real world and is widely used in ontological design. The DCPO was merged with 2 top-level ontologies—Basic Formal Ontology and Ontology for General Medical Science—in a method of merging top-level ontologies that are often used by researchers [30-32]; it has been shown to facilitate the reuse of terms from existing ontologies constructed under top-level ontologies [33].

#### Stage 2: DCPO Model Initialization

In the second stage, class attributes are defined to describe the internal structure of concepts because the class hierarchy is insufficient to distinguish the relationships between concepts. These attributes include object attributes that describe the relationship between 2 individuals and data attributes that describe the relationship between an individual and a data value [34,35]. The precise semantics of restricting classes by adding attributes is accomplished, and these restrictions are expressed as a set of axioms. These axioms include property axioms that describe aspects of properties such as domain and range and individual axioms that describe anonymous individual classes. The instances of each class are created in the hierarchy. The core part of an instance is a class called the patient profile. Related feature instances are created and bound to the patient profile instance for further rule-based reasoning.

#### Stage 3: Rule Definition

In the third phase, external semantic rules are used to implement the complex deductive reasoning required for path-driven decision support. In this study, the SWRL rules are used to incorporate the medical knowledge of the pathway model. The input and output of the rule and the state representation of the reasoning are defined. The inputs and outputs of the rules vary significantly for different pathway tasks. On the basis of the basic DCPO and predefined SWRL rule sets, various pathway tasks will be generated based on raw patient data and then converted into executable management plans, including physician intervention plans and patient self-management plans. This is a crucial part of the knowledge-based clinical decision support system engines.

### System Deployment

A DCPO-based closed-loop diabetes management system was designed and applied to evaluate the practical applications of the research results. The T2DMMS is involved in a diabetes management service scenario to achieve comprehensive management and intervention guidance for patients with type 2 diabetes.

#### System Framework Design

**Figure 3:**
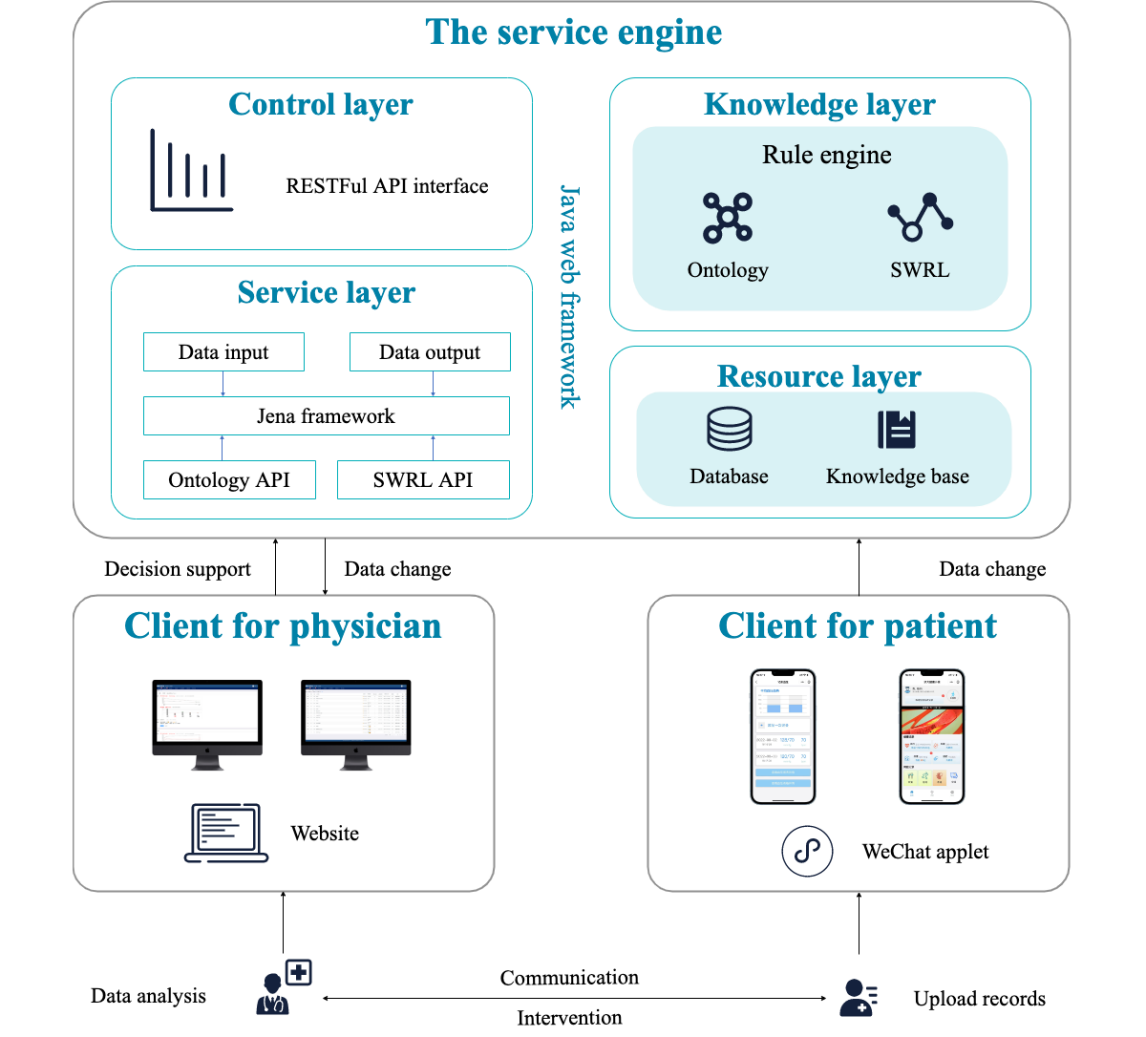
The Type 2 Diabetes Mellitus Management System architecture. API: application program interface; REST: representational state transfer; SWRL: Semantic Web Rule Language.

The architecture of the T2DMMS is shown in Figure 3, which includes 3 parts as follows: an intelligent service engine, a physician-oriented client, and a patient-oriented client. The service engine runs on the cloud server and is the core module of T2DMMS. Its primary function is to integrate the ontology knowledge base with the SWRL rule base and patient data; it also plays a role in realizing logical reasoning and providing the web service interface to interact with physician-oriented clients and realize decision support based on the T2DMMP. Physician-oriented clients are mainly responsible for displaying patients’ health data. Physicians can complete a comprehensive assessment of patients by viewing the data and complete management tasks by providing intervention guidance and improving patient compliance efficiently. For patient-oriented clients, patients can view their self-management plans and complete their daily management tasks by uploading their health data records. In addition, patients can communicate with their physicians through their clients.

#### Development Tools

The intelligent service engine is based on the SpringBoot+MyBatis+MySQL design architecture. The presentation form on the physician-oriented client is a Webpage and WeChat Mini Program (Tencent). The patient-oriented client is mainly the WeChat Mini Program.

### Retrospective Evaluation

As the initial dates of patients’ inclusion in the closed-loop diabetes management system for management were different, the date of each patient’s inclusion in the management was used as the start of the study, whereby the management data for the following 3 months were recorded. If the management period was longer than 3 months, only the data for the first 3 months were collected for analysis. All patient data in this study were analyzed statistically using SPSS software (version 24.0; IBM Corp). The continuous assessment indexes were analyzed for significant differences in the management process using Student’s *t* test (2-tailed), and the assessment indexes for the attainment rate were analyzed using the chi-square test to determine whether the data had significant differences.

The study involved 272 patients with diabetes who were included in the T2DMMS for typical management from January 2020 to August 2020 in the Ning-xia Medical University General Hospital Group. The patient inclusion criteria were as follows: (1) patients who signed informed consent form for the trial, (2) patient whose management time >3 months, and (3) patients whose BP and BG recorded >3 times. The patient exclusion criteria were as follows: (1) patients whose BG was not recorded after inclusion in management, (2) patients who were lost to follow-up, (3) patients with complex and severe comorbidities, and (4) patients with mental cognitive or physical dysfunction.

### Ethics Approval

All the patients who entered the telehealth system signed an informed consent form. The nursing staff also signed informed consent forms. All the procedures were conducted according to the ethical guidelines for biomedical research involving humans at Ning-xia Medical University. Ethics approval was granted by the Ethics Committee for the Conduct of Human Research at the General Hospital of Ning-xia Medical University (NXMU-GH-2017-273).

## Results

### Ontology Construction and Evaluation

**Figure 4:**
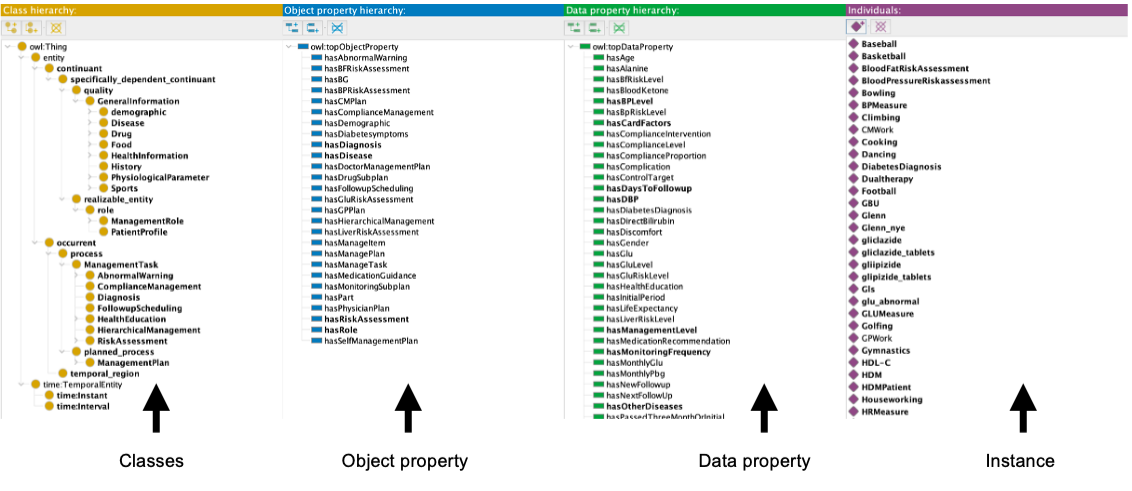
The snapshot of Diabetes Care Pathway Ontology (DCPO) from the Protégé 5.5.0.

The DCPO was developed in the Web Ontology Language file format by the ontology editor Protégé 5.5.0. A snapshot of the DCPO is shown in Figure 4. The newest version of the DCPO contains 119 newly added classes, 28 object properties, 58 data properties, 81 individuals, and 426 axioms. In addition, 192 SWRL rules were newly added to implement the diabetes management care pathway. The SWRL rules are divided into 10 modules, with 22.9% (44/192) of rules based on clinical expert experience, 43.2% (83/192) based on medical guidelines, and 33.9% (65/192) based on both clinical expert experience and medical guidelines. Table 1 presents the specific distributions. The complete SWRL is presented in Multimedia Appendix 1.

**Figure 5:**
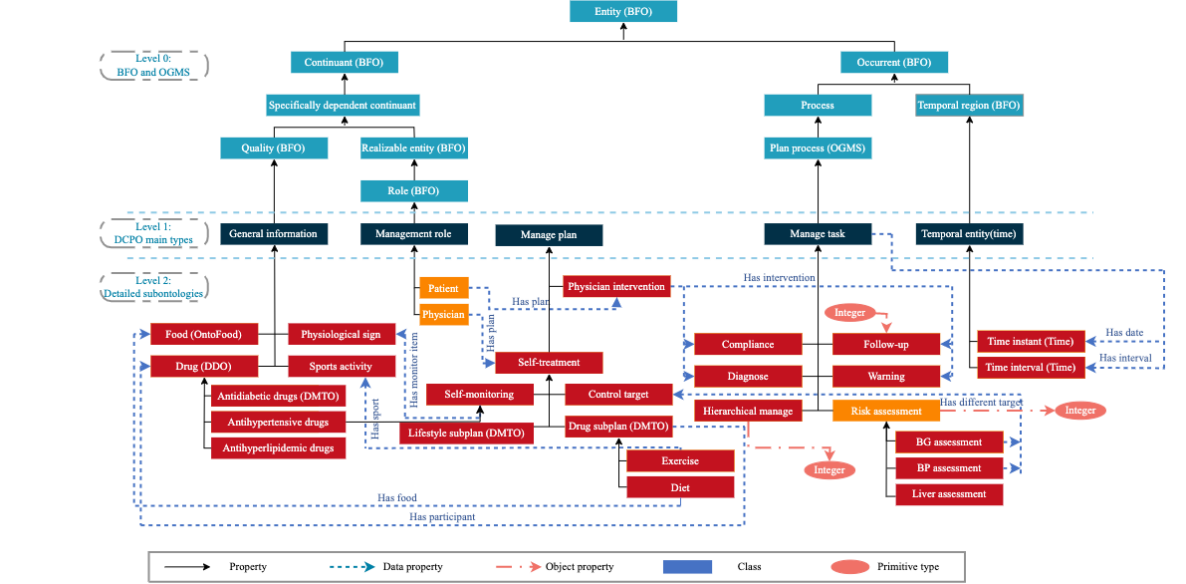
The class diagram of Diabetes Care Pathway Ontology (DCPO)’s main core. BFO: Basic Formal Ontology; BP: blood pressure; BG: blood glucose; DMTO: Diabetes Mellitus Treatment Ontology; OGMS: Ontology for General Medical Science.

The class diagram of the main core of the DCPO is shown in Figure 5. The DCPO mainly consists of the following 3 levels: level 0 includes several common top-level ontologies, which are regarded as standard to implement and improve the interoperability of other ontologies; level 1 consists of 5 terms that described the core concepts in the diabetes management pathway model; and level 2 is the detailed elements for each level-1 term.

First, the patient profile class is used to generate the instance of the patient condition through the object properties to connect to other main class instances. The management task represented the main content of the diabetes management pathway model, and it was synergistic and sequential, covering the whole process of diabetes diagnosis and treatment. In addition, the DCPO can generate management tasks with different contents for patients with different characteristics using instances and a set of SWRL rules. The content of the generated management tasks is converted into management plans.

**Table 1:** Semantic Web Rule Language rule construction results.

Rules module	Derived from clinical expert experience (n=44), n (%)	Derived from medical guidelines (n=83), n (%)	Derived from both clinical expert experience and medical guidelines (n=65), n (%)
Diagnosis patterns	0 (0)	10 (12)	0 (0)
Risk assessment	11 (25)	12 (14)	28 (43)
Control objectives	0 (0)	6 (7)	3 (5)
Hierarchical management	18 (41)	0 (0)	0 (0)
Self-monitoring	0 (0)	0 (0)	18 (28)
Regular follow-up	0 (0)	0 (0)	8 (12)
Abnormal attention	15 (34)	0 (0)	0 (0)
Medication guidance	0 (0)	45 (54)	0 (0)
Lifestyle guidance	0 (0)	10 (12)	3 (5)
Compliance management	0 (0)	0 (0)	5 (8)

### System Interaction

The physician-oriented client of the T2DMMS is operated by the medical service provider on the web page, and the patient-oriented client is displayed in the form of a WeChat applet. The 2 clients are coordinated by the diabetes management path in the intelligent service engine to efficiently complete the management work and improve patient compliance.

**Figure 6:**
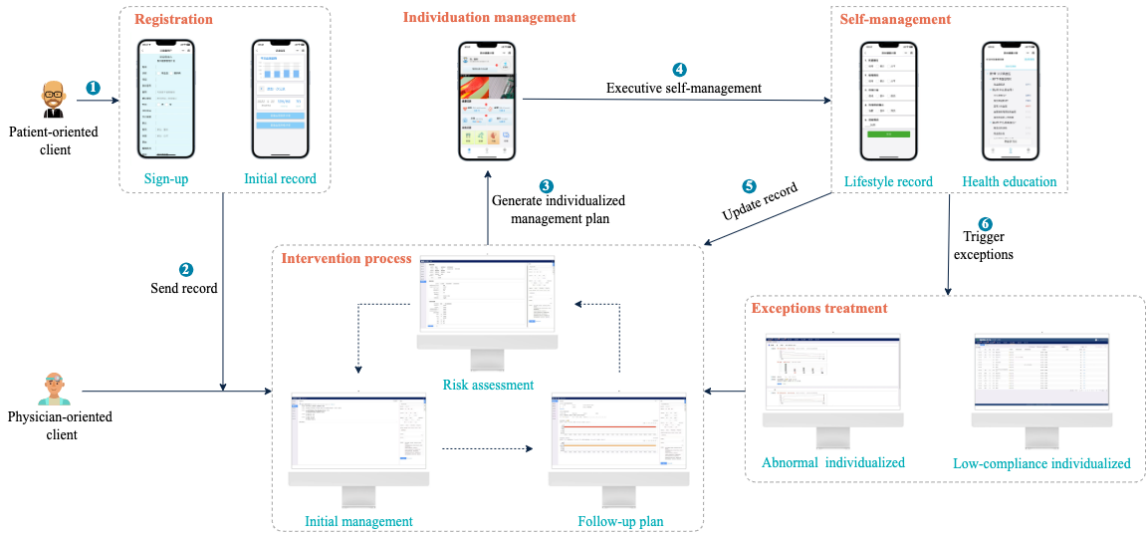
Schematic of system interaction.

As shown in Figure 6, the physician-oriented client assists physicians in diagnosis and treatment tasks, including risk assessment, initial management, regular follow-up, abnormal attention, and compliance management for patients. A closed-loop path is formed between risk assessment, initial management, and follow-up, which can provide long-term management for patients. The physician only needs to operate in a certain order on the client terminal to manage the patient. In addition, the physician-oriented clients can modify the process, timing, and content of physician interventions to guide patients in the diabetes management path.

The patient-oriented client receives the personalized management plan analyzed by the intelligent service engine and displays the corresponding generated individualized management plan to the patients in the form of daily tasks, reminding the patient to follow the physician’s instructions to complete the management plan. According to the prompts of daily tasks, patients can record their own health data every day through the WeChat applet; they can not only obtain real-time feedback and intervention from the engine but also receive feedback and intervention guidance from physicians.

Patient health data records mainly include BG levels, BP levels, discomfort symptom, weight, diet, and medication guidance records. The patient’s health data records will be used as the data input for the intelligent service engine to evaluate and analyze the patient. When an abnormal attention occurs, the engine will provide real-time intervention guidance push and send the emergency situation of the patient to the physician to remind the physician to intervene.

### Retrospective Study Data

#### Overview

In this study, 272 patients with a mean age of 58.24 (SD 9.81) years were selected, of which 156 (57.4%) patients had diabetes only, and 116 (42.6%) patients had both hypertension and diabetes. According to the guidelines’ recommendations, the initial BG levels were assessed in all 100% (272/272) of patients with diabetes. The BG levels were normal in 34.9% (95/272) of patients and abnormal in 65.1% (177/272) of patients. The initial BP was also assessed in 42.6% (116/272) of patients with hypertension and diabetes. The BP was normal in 30.2% (35/116) of patients and abnormal in 69.8% (81/116) of patients. Detailed patient data are presented in Table 2.

The management data of all patients were extracted from the T2DMMS during the 3-month management period. The data were analyzed from the following 2 perspectives: the investigation of physician work and analysis of patients’ indicators.

**Table 2:** Experimental subject details (N=272).

Characteristics			Value
**Sex, n (%)**			
	Male	147 (54)	
	Female	125 (46)	
**Disease type, n (%)**			
	Diabetes only	156 (57.4)	
	Both diabetes and hypertension	116 (42.6)	
**Initial assessment, n (%)**			
	Normal blood glucose level	95 (34.9)	
	Abnormal blood glucose level	177 (65.1)	
	Normal blood pressure^a^	35 (30.2)	
	Abnormal blood pressure^a^	81 (69.8)	
Age, mean (SD) years			58.24 (9.81)
**Special groups, n (%)**			
	Adolescent	7 (2.5)	
	Disabilities	2 (0.7)	

^a^n=116, which is the number of patients with hypertension.

#### Investigating Physician Work

**Figure 7:**
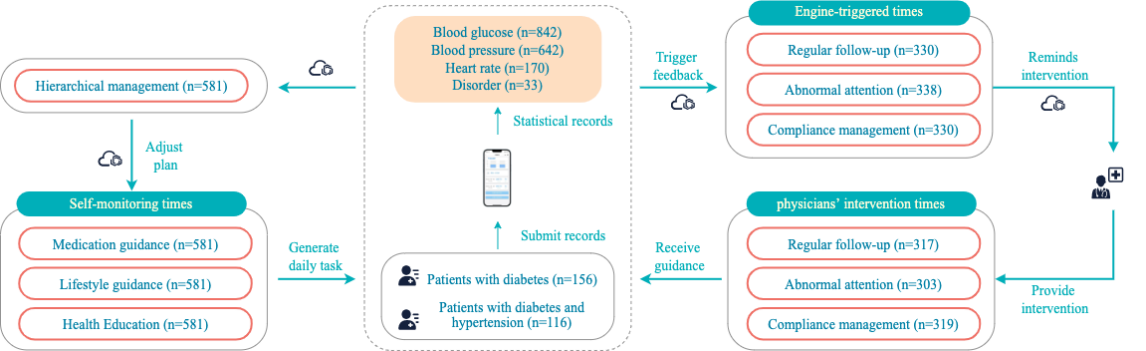
Overall management records of patients and physicians during the 3-month period.

Figure 7 shows overall intervention records and patients’ self-monitoring data through the engine and system following the diabetes management care pathway. Compared with the usual patient management approach based on guidelines only, T2DMMP added abnormal data attention and low-compliance management. As shown in Table 3, during the 3-month management cycle, the physician provided 904 follow-up visits in the usual patient management approach based on guidelines, and they followed up patients 939 times in T2DMMP management system, including 317 (33.8%) regular follow-up visits; 303 (32.3%) follow-up visits caused by abnormal attention that included 75 (24.8%) BG warning, 150 (49.5%) BP warning, 33 (10.9%) disorder warning, and 45 (14.9%) heart rate warning, and 319 (34%) follow-up visits for low-compliance management. The results showed that not only the physician’s work contents were refined but also the number of follow-ups by the physician were changed in the T2DMMP-based management approach. The changes improved the attention and number of interventions for patients with poorer conditions.

The physician had not fully completed the intervention plan because some of the warnings were repeated, and the regular follow-up plan of patients who had hypertension and diabetes was incorporated.

The actual number of follow-up visits was 939 by physicians based on pathway prompts. Of the 272 patients, the number of follow-up visits was 855 (91.1%) in 242 (89%) patients whose initial BG level or BP was abnormal, and each patient was followed up 3.53 (SD 1.07) times on average. The number of follow-up visits was 84 (18.9%) in 30 (11%) patients whose initial BG level or BP was normal, and each patient was followed up with 2.8 (SD 0.59) times on average. Of the 855 follow-up visits owing to initial abnormalities in BG or BP, the real number of follow-up visits made by physicians to patients was classified into the following 3 levels: the number of follow-up visits >3 times was defined as high-intervention level; the number of follow-up visits equal to 3 times was defined as medium-intervention level; and the number of follow-up visits <3 times was defined as low-intervention level. Of the 242 patients with 855 interventions, there were 55 (22.7%) patients with high-intervention level, (398/855, 46.5%) and an average of 7.2 follow-ups per patient; there were 30 (12.4%) patients with medium-intervention level (154/855, 18%) and an average of 3.1 (SD 0.67) follow-ups per patient, 157 (64.9%) patients with low-intervention level (303/855, 35.4%), and an average of 1.9 (SD 1.25) follow-ups per patient. The guidelines state that physicians should follow-up at least once a month for patients with substandard BG, so physicians should follow-up each substandard patient 3 times within a 3-month management cycle. The above analysis results showed that a series of work can be dynamically adjusted according to the actual condition control of patients in the diabetes management pathway constructed in this study, including the follow-up schedule, prompting physicians to give different interventions and attention to patients with different management statuses. The adjusted changes can help patients whose condition control is poor obtain limited medical resource services more efficiently. Diabetes management pathways can improve physicians’ working efficiency compared with the management style of managing patients based on guidelines only.

**Table 3:** Comparison of the number of follow-up visits to patients with different management models.

	Follow-up visits using management models	
	Based on diabetes management guidelines (n=904), n (%)	Based on the diabetes management care pathway (n=939), n (%)
Regular follow-up	904 (100)	317 (33.8)
Abnormal attention	0 (0)	303 (32.3)
Compliance management	0 (0)	319 (34)

#### Analysis of Patient Indicators

**Figure 8:**
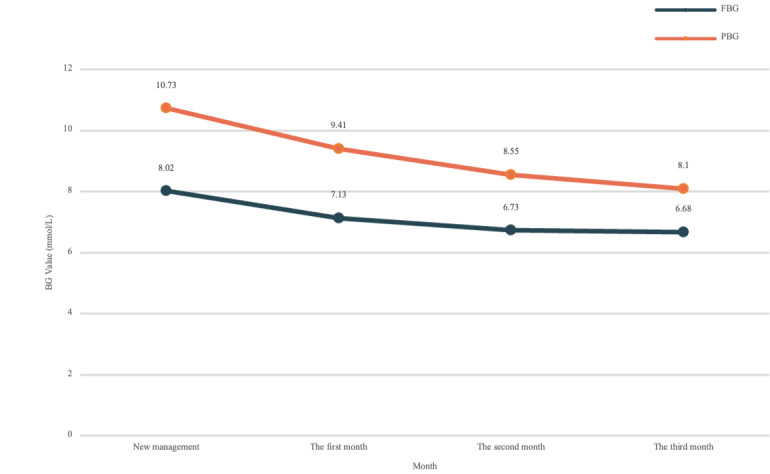
The records of patient’s blood glucose changes during the 3-month management period. BG: blood glucose; FBG: fasting blood glucose; PBG: postprandial blood glucose.

**Figure 9:**
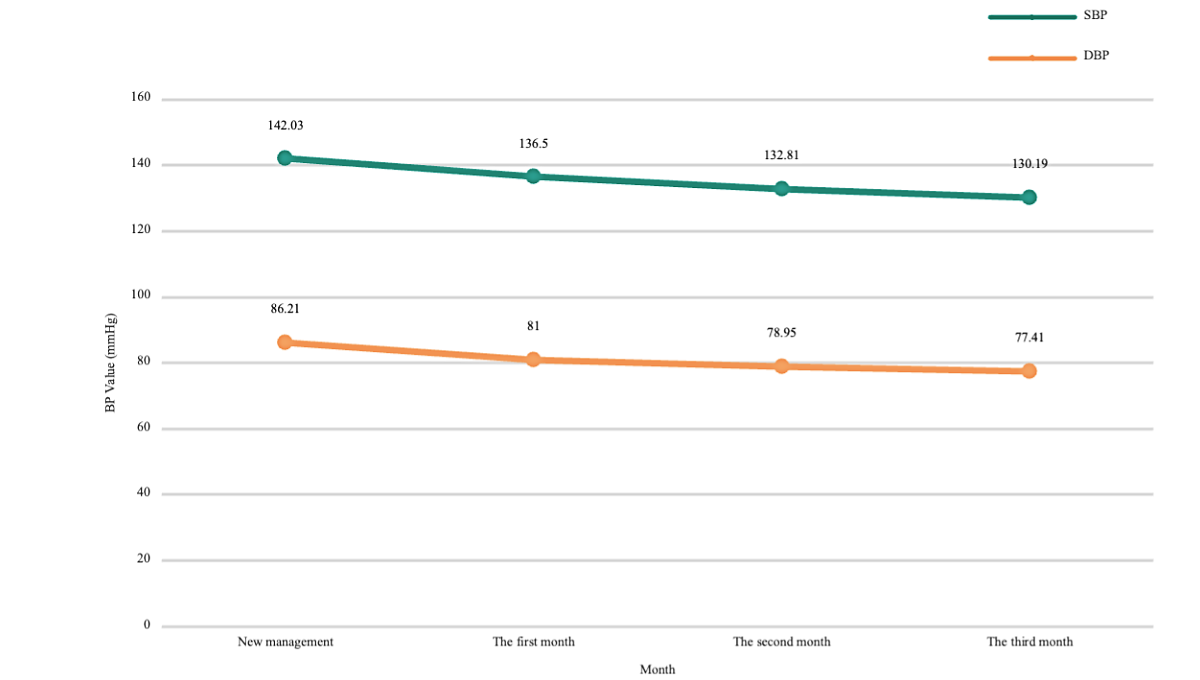
The records of patient’s blood pressure changes during the 3-month management period. BP: blood pressure; DBP: diastolic blood pressure; SBP: systolic blood pressure.

The core goal of this study was to achieve effective management of patients with diabetes and comprehensive management of multiple cardiovascular risk factors. Whether the patients’ BG or BP decrease is the key metric to evaluate the application effect of the diabetes management model and DCPO designed in this study. The BG data records, which were uploaded by patients, were analyzed in all 100% (272/272) of patients during the 3 months, including FBG and postprandial BG (PBG). In addition, BP data (systolic BP [SBP] and diastolic BP [DBP]) were analyzed in 116 (42.6%) patients with hypertension and diabetes. All BG and BP data were obtained from the T2DMMS database. The FBG level of patients was recorded 2 hours before meals, and the PBG was recorded 2 hours after meals. The patients’ BP was recorded between 8 AM and 10 AM. We analyzed the mean trend of all patients over the 3-month management period. Figures 8 and 9 show the patients’ monthly average BG and BP records during the 3-month management period. Figure 8 showed the monthly mean FBG level decreased by 1.34 mmol/L (*P*=.003) and the monthly mean PBG level decreased by 2.63 mmol/L (*P*=.003) in all (272/272, 100%) patients with diabetes during the 3-month management period. Figure 9 shows that the monthly mean BP level also decreased significantly and finally reached a stable level in all (116/272, 42.6%) patients with both diabetes and hypertension during the 3-month management period in which the monthly mean SBP decreased by 11.84 mmHg (*P*=.02), and the monthly mean DBP decreased by 8.8 mmHg (*P*=.02).

**Figure 10:**
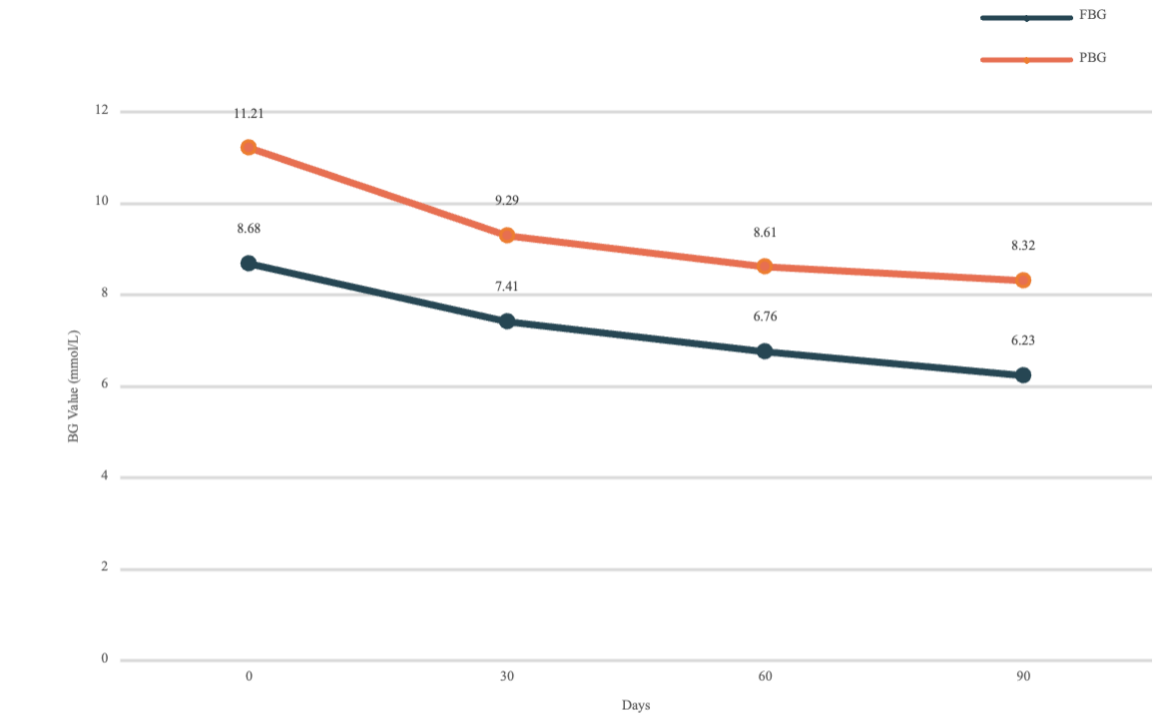
The records of intervened patient’s blood glucose during the 3-month management period. BG: blood glucose; FBG: fasting blood glucose; PBG: postprandial blood glucose.

**Figure 11:**
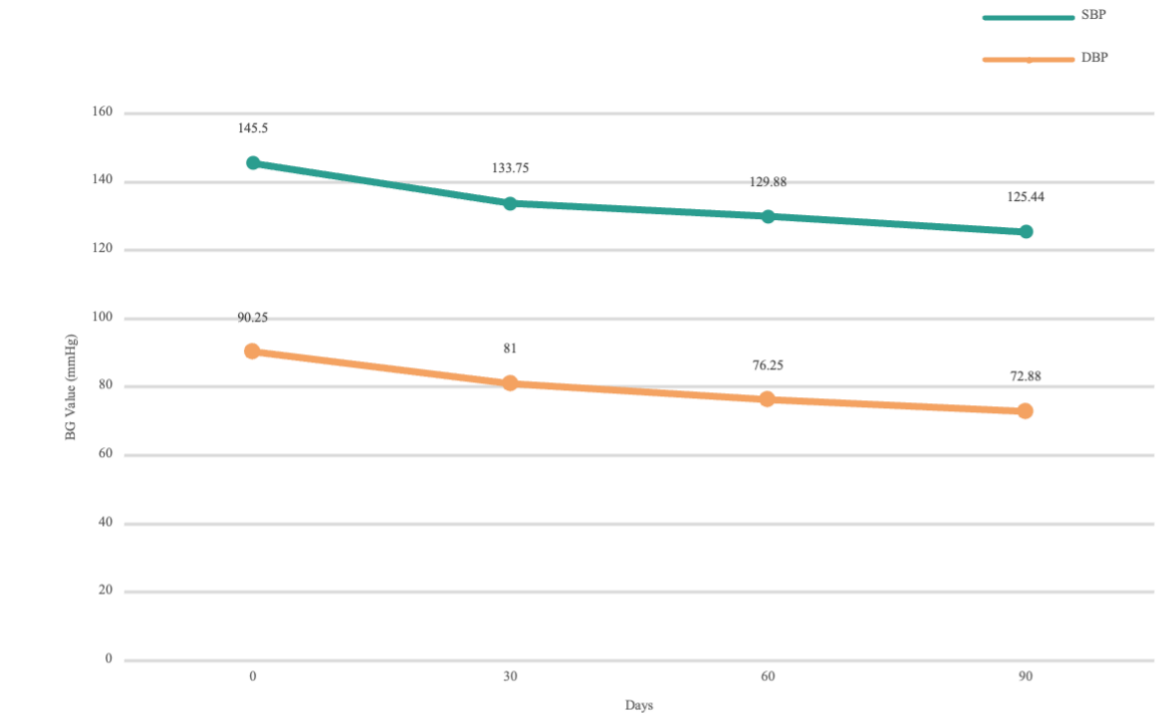
The records of intervened patient’s blood pressure during the 3-month management period. BG: blood glucose; DBP: diastolic blood pressure; SBP: systolic blood pressure.

We screened patients who received physician interventions during the management cycle owing to abnormal attention or low-compliance warnings from the trigger system. In patients with diabetes only, trends in FBG and PBG levels were analyzed. For patients with both hypertension and diabetes, trends in SBP and DBP were analyzed. As can be seen from the statistics in Figures 10 and 11, the mean monthly BG and BP values of the patients decreased significantly with respect to additional physician interventions. The monthly mean FBG level decreased by 2.45 mmol/L (*P*=.003) and the monthly mean PBG level decreased by 2.89 mmol/L (*P*=.003) in all (272/272, 100%) patients with diabetes during the 3-month management period; the monthly mean SBP decreased by 20.06 mmHg (*P*=.02) and the monthly mean DBP decreased by 17.37 mmHg (*P*=.02) in patients (116/272, 42.6%) with both hypertension and diabetes during the 3-month management period.

The analysis of the above results proves that the DCPO constructed in this study positively stabilizes the patient’s condition by defining the management responsibilities corresponding to different management roles and adding the physician’s intervention plan. It can also effectively help physicians manage patients with diabetes comprehensively.

## Discussion

### Principal Findings

In this study, we proposed and constructed the T2DMMS for monitoring and managing patients with type 2 diabetes. The T2DMMS could realize the comprehensive management and physician-patient interaction and intervention by defining different management roles corresponding to different management responsibilities proposed and defined in the diabetes management pathway. In addition, we implemented a telehealth system based on the T2DMMS and applied it to actual diabetes management. According to the retrospective analysis of the patient profile data records, the T2DMMS could realize the comprehensive evaluation and management of patients with diabetes and positively impact the comprehensive indicators of patients. Patients can self-manage according to the diabetes management care pathway and receive intervention guidance from physicians. The results indicated that the comprehensive indicators of patients certainly improved after the intervention.

To construct the T2DMMS, we first designed and implemented the diabetes management care pathway (T2DMMP) model. We summarized the diabetes management process into 9 core management tasks based on the CDMP concept and integrated these into a sequential and closed-loop diabetes management pathway model. Meanwhile, we defined the different management roles of physicians and patients, clarified their respective responsibilities, and realized an excellent physician-patient interaction intervention mechanism. Then, we referred to the top-down ontology construction method to construct the DCPO and realized the digitization of the T2DMMP expression process and construction results. By combining the top-level ontology and the existing standard ontology, we defined the main terms of the DCPO as subclasses of these top-level ontologies and finally built the DCPO with a 3-level hierarchy. To achieve more complete diabetes management, we formulated the SWRL rules in the standard coding method to achieve the timing between the management tasks in the T2DMMP.

From the system’s retrospective evaluation results, we identified several aspects and features. First, the monthly mean BG levels of all patients under regular management showed a downward trend. For patients with diabetes and hypertension, the monthly mean BP levels also decreased substantially. Second, the work content of physicians was defined by the diabetes management care pathway, including regular follow-up, abnormal warning, and compliance follow-up intervention. The system will remind physicians to implement interventional guidance. The data analysis showed that patients who had received additional interventions such as abnormal warning or compliance intervention had a more significant reduction in monthly mean BG and BP than patients who only received intervention guidance from regular follow-up.

### Comparison With Previous Work

Table 4 provides a comparison between the DCPO and 3 other diabetes management ontologies based on the 10 dimensions. As shown in the table, all 3 compared ontologies were limited to patients’ comprehensive diabetes management, and the management plan mainly focused on patients, ignoring the responsibility of physicians and importance of physicians’ intervention. The DCPO can standardize and guide the comprehensive and complete management of patients with diabetes in primary care institutions in China. In addition, the DCPO performs well in terms of reusability, extensibility, and semantic interoperability.

Compared with previous work, our study is considered innovative in the following aspects: (a) For management, we innovatively introduced the concept of the pathway and then combined it with the information system to establish a standardized and executable management model. This system provides a one-stop platform for physicians and a fully functional terminal for patients. Physicians can perform almost all the daily work on the platform, and patients are able to monitor their BP and BG and receive management advice from their physicians. These 2 clients are connected by an engine that provides automatic decision support during the management process. To the best of our knowledge, such a fully functional and highly usable system for diabetes management in China has not yet been reported in the literature. (b) For the trial design, we proposed 2 perspective outcome measures as follows: physician work content analysis and control of comprehensive patient indicators. Controlling comprehensive patient indicators is an effective treatment for patients with diabetes.

**Table 4:** Comparison of Diabetes Care Pathway Ontology and other diabetes treatment ontologies.

Dimension	DCPO^a^	DMTO^b^	OMDP^c^	DDO^d^
Purpose	T2DM^e^ treatment	T2DM treatment	T2DM treatment	T2DM treatment
Based on top-level ontology	Yes	Yes	Yes	Yes
Integration of the pathway tasks	Diagnosis, risk assessment, hierarchical management, regular follow-up, abnormal warning, medication guidance, lifestyle guidance, health education, and compliance management	Diagnosis and treatment by drug, food, exercise, and education	Prognosis, diagnosis, and treatment plan	Diagnosis
Treatment decision-making	Based on the risk results, management level, and past and current index of patient	Based on the patient’s whole profile, including laboratory tests	Based on the patient’s laboratory test results	Based on the patient’s blood glucose and other laboratory tests
Modeling temporal semantics	Yes	Yes	No	No
Modeling comorbidities and complications	Hypertension, hyperlipidemia, hypoglycemia, and hyperglycemia	Diabetic nephropathy, retinopathy, and other complications	No clear definition	Diabetic ketoacidosis and coronary heart disease
Model of management roles	Defining both physician and patient	No management role defined	No management role defined	No management role defined
Application in telehealth environments	Supported by an intelligent service engine	No application	No application	No application
Drug modeling in the ontology	Antidiabetes drugs, hypertension drugs, and lipid drugs	Antidiabetes drugs, diabetes complication drugs, and the drug-drug interactions	Antidiabetes drugs and other drugs used for complications	Drugs affecting blood glucose
SWRL^f^ rule sources in the ontology	Combining clinical experts and medical guidelines	No SWRL rules	Separate use of medical guidelines	No SWRL rules

^a^DCPO: Diabetes Care Pathway Ontology.

^b^DMTO: Diabetes Mellitus Treatment Ontology.

^c^OMDP: Ontology-Based Model for Diagnosis and Treatment of Diabetes Patients.

^d^DDO: Diabetes Diagnosis Ontology.

^e^T2DM: type 2 diabetes mellitus.

^f^SWRL: Semantic Web Rule Language.

### Conclusions

In this study, a diabetes management pathway model was constructed, a diabetes management ontology for comprehensive diabetes management was developed to achieve physician-patient intervention, and a telehealth system based on this ontology was developed by summarizing the important process and core content of diabetes management. The DCPO was constructed on the basis of the general semantic definition of the standard top-level ontology, which contained 119 newly added classes, 28 object properties, 58 data properties, 81 individuals, 426 axioms, and 192 SWRL rules; this covered the entire process of diabetes management and managed multiple cardiovascular risk factors for patients with diabetes. Further research should be considered to deal with the ambiguity of medical semantics using fuzzy ontology; enhance the accuracy and reasoning ability of the system; introduce data-driven technology, considering the semantic interoperability with the electronic health record system; and obtain more clinical information from patient information to achieve a more personalized management plan.

## Appendix

Multimedia Appendix 1 Semantic Web Rule Language rules for Diabetes Care Pathway Ontology.

## Abbreviations

BG: blood glucose
BP: blood pressure
CDMP: Chronic Disease Management Pathway
DBP: diastolic blood pressure
DCPO: Diabetes Care Pathway Ontology
FBG: fasting blood glucose
PBG: postprandial blood glucose
SBP: systolic blood pressure
SWRL: Semantic Web Rule Language
T2DM: type 2 diabetes mellitus
T2DMMP: Type 2 Diabetes Mellitus Management Pathway
T2DMMS: Type 2 Diabetes Mellitus Management System
