# Supplementary material for: Telehealth System Based on the Ontology Design of a Diabetes Management Pathway Model in China: Development and Usability Study
Source: JMIR Med Inform. 2022 Dec 19;10(12):e42664. doi: 10.2196/42664 (PMC9808585; doi:10.2196/42664)
Supplement: Multimedia Appendix 1 [file medinform_v10i12e42664_app1.docx]

**Appendix 1**

Table 1 Expression results of SWRL rules for diabetes diagnosis

| serial | rules | rules definition | source |
| --- | --- | --- | --- |
| D101 | Normal blood glucose-1 | PatientProfile(?p)^hasBG(?p,?bg)^hasGlu(?p,?g)^swrlb:lessThan(?g,"6.1"^^xsd:float) ->NormalDiabetes(? bg) | Guidelines for the prevention and control of type 2 diabetes in China  Page:294 Table 2 |
| D102 | Normal blood glucose-2 | PatientProfile(?p)^hasBG(?p,?bg)^hasPBG(?p,?pg)^swrlb:lessThan(?pg,"7.8"^^xsd:float)->NormalBloodGlucose(? bg) | Guidelines for the prevention and control of type 2 diabetes in China  Page:294 Table 2 |
| D103 | impaired fasting glucose | PatientProfile(?p)^hasBG(?p,?bg)^hasGlu(?p,?g)^swrlb:lessThan(?g,"7.0"^^xsd:float)^swrlb:greaterThanOrEqual(?g,"6.1"^^xsd:float) ->Impaired_Glucose_Regulation(? bg) | Guidelines for the prevention and control of type 2 diabetes in China  Page:294 Table 2 |
| D104 | impaired glucose tolerance | PatientProfile(?p)^hasBG(?p,?bg)^hasPBG(?p,?pg)^swrlb:lessThan(?pg,"11.1"^^xsd:float)^swrlb:greaterThanOrEqual(?g,"7.8"^^xsd:float) ->Impaired_Glucose_Regulation(? bg) | Guidelines for the prevention and control of type 2 diabetes in China  Page:294 Table 2 |
| D105 | Abnormal blood glucose-1 | PatientProfile(?p)^hasDiabetesymptoms(?p,?s)^hasThreeSymptoms(?s,false)^hasWeightLow(?s,fasle)^hasBG(?p,?bg)^hasPBG(?p,?g)^swrlb:greaterThanOrEqual(?g,"11.1"^^xsd:float) ->hasGluLevel(? bg,glu_abnormal) | National guidelines for the prevention and control of diabetes in primary care  Page :887 table 1 |
| D106 | Abnormal blood glucose-2 | PatientProfile(?p)^hasDiabetesymptoms(?p,?s)^hasThreeSymptoms(?s,false)^hasWeightLow(?s,fasle)^hasBG(?p,?bg)^hasGlu(?p,?g)^swrlb:greaterThanOrEqual(?g,"7.0"^^xsd:float) ->hasGluLevel(? bg,glu_abnormal) | National guidelines for the prevention and control of diabetes in primary care  Page :887 table 1 |
| D107 | Abnormal blood glucose-3 | PatientProfile(?p)^hasDiabetesymptoms(?p,?s)^hasThreeSymptoms(?s,false)^hasWeightLow(?s,fasle)^hasBG(?p,?bg)^hasRandomGlu(?p,?g)^swrlb:greaterThanOrEqual(?g,"11.1"^^xsd:float) ->hasGluLevel(? bg,glu_abnormal) | National guidelines for the prevention and control of diabetes in primary care  Page :887 table 1 |
| D108 | Diabetes-1 | PatientProfile(?p)^hasDiabetesymptoms(?p,?s)^hasThreeSymptoms(?s,true)^hasWeightLow(?s,true)^hasBG(?p,?bg)^hasGlu(?p,?g)^swrlb:greaterThanOrEqual(?g,"7.0"^^xsd:float) ->Diabetes(? p) | Guidelines for the prevention and control of type 2 diabetes in China  Page:294 Table 2 |
| D109 | Diabetes-2 | PatientProfile(?p)^hasDiabetesymptoms(?p,?s)^hasThreeSymptoms(?s,true)^hasWeightLow(?s,true)^hasBG(?p,?bg)^hasPBG(?p,?g)^swrlb:greaterThanOrEqual(?g,"11.1"^^xsd:float) ->Diabetes(? p) | Guidelines for the prevention and control of type 2 diabetes in China  Page:294 Table 2 |
| D110 | Diabetes-3 | PatientProfile(?p)^hasDiabetesymptoms(?p,?s)^hasThreeSymptoms(?s,true)^hasWeightLow(?s,true)^hasBG(?p,?bg)^hasRandomGlu(?p,?g)^swrlb:greaterThanOrEqual(?g,"11.1"^^xsd:float) ->Diabetes(? p) | Guidelines for the prevention and control of type 2 diabetes in China  Page:294 Table 2 |

Table 2 Expression results of SWRL rules for diabetes comprehensive risk assessment

| serial | rules | rules definition | source |
| --- | --- | --- | --- |
| D201 | Aged over 60 years without complications | hasDiabetesDiagnosis(?p,true)^PatientProfile(?p)^hasManageTask(?p,?m)^hasRiskAssessment(?m,?r)^hasGluRiskAssessment(?r,?g)^hasAge(?r,?a)^swrlb:greaterThanOrEqual(?a,"60"^^xsd:integer)^hasComplication(?r,false)-> hasGluRiskLevel(?g, "2"^^xsd:integer) | Both Clinical expert experience and medical guidelines |
| D202 | Aged over 75 | hasDiabetesDiagnosis(?p,true)^PatientProfile(?p)^hasManageTask(?p,?m)^hasRiskAssessment(?m,?r)^hasGluRiskAssessment(?r,?g)^hasAge(?g,?a)^swrlb:greaterThan(?a,"75"^^xsd:integer)->hasGluRiskLevel(?g,"3"^^xsd:integer) | Both Clinical expert experience and medical guidelines |
| D203 | Aged older than 60 and have complications | hasDiabetesDiagnosis(?p,true)^PatientProfile(?p)^hasManageTask(?p,?m)^hasRiskAssessment(?m,?r)hasGluRiskAssessment(?r,?g)^hasAge(?r,?a)^swrlb:greaterThanOrEqual(?a,"60"^^xsd:integer)^hasComplication(?r,true)->hasGluRiskLevel(?g,"3"^^xsd:integer) | Both Clinical expert experience and medical guidelines |
| D204 | life expectancy less than 5 years | hasDiabetesDiagnosis(?p,true)^PatientProfile(?p)^hasManageTask(?p,?m)^hasRiskAssessment(?m,?r)^hasGluRiskAssessment(?r,?g)^hasLifeExpectancy(?g, ?e)^swrlb:lessThan(?e,"5"^^xsd:integer)->hasGluRiskLevel(?g, "3"^^xsd:integer) | Clinical expert experience |
| D205 | younger than 60 with complications | hasDiabetesDiagnosis(?p,true)^PatientProfile(?p) ^hasManageTask(?p,?m)^hasRiskAssessment(?m,?r)^hasGluRiskAssessment(?r,?g)^hasAge(?r, ?a)^swrlb:lessThan(?a,"60"^^xsd:integer)^hasComplication(?r,true)->hasGluRiskLevel(?g,"2"^^xsd:integer) | Both Clinical expert experience and medical guidelines |
| D206 | Life expectancy within 5 to 15 years and age less than 60 | hasDiabetesDiagnosis(?p,true)^PatientProfile(?p)^hasManageTask(?p,?m)^hasRiskAssessment(?m,?r)^hasGluRiskAssessment(?r,?g)^hasLifeExpectancy(?r,?e)^swrlb:lessThanOrEqual(?e,"15"^^xsd:integer)^swrlb:greaterThanOrEqual(?e,"5"^^xsd:integer)^hasAge(?r,?a)^swrlb:lessThan(?a,"60"^^xsd:integer)^hasComplication(?r,false)->hasGluRiskLevel(?g,"1"^^xsd:integer) | Clinical expert experience |
| D207 | obesity with substandard blood glucose | hasDiabetesDiagnosis(?p,true)^PatientProfile(?p)^hasManageTask(?p,?m)^hasRiskAssessment(?m, ?r)^hasGluRiskAssessment(?r,?g)^hasOverWeight(?g,true)^hasGluLevel(?g,glu_abnormal)->hasGluRiskLevel(?g,"2"^^xsd:integer) | Guidelines for the prevention and control of type 2 diabetes in China  Page:296 paragraph:4.1 |
| D208 | substandard blood glucose | hasDiabetesDiagnosis(?p,true)^PatientProfile(?p)^hasManageTask(?p,?m)^ hasRiskAssessment(?m, ?r)^ hasGluRiskAssessment(?r,?g)^hasOverWeight(?g,fasle)^hasGluLevel(?g,glu_abnormal)-> hasGluRiskLevel(?g, "1"^^xsd:integer) | Guidelines for the prevention and control of type 2 diabetes in China  Page:296 paragraph:4.2 |
| D209 | standard blood glucose with normal BMI | hasDiabetesDiagnosis(?p, true) ^ PatientProfile(?p) ^hasManageTask(?p,?m)^ hasRiskAssessment(?m, ?r)^ hasGluRiskAssessment(?r,?g)^hasOverWeight(?g,fasle)^hasGluLevel(?g,glu_normal)-> hasGluRiskLevel(?g, "1"^^xsd:integer) | Guidelines for the prevention and control of type 2 diabetes in China  Page:296 paragraph:4.2 |
| D210 | obesity | hasDiabetesDiagnosis(?p, true) ^ PatientProfile(?p) ^hasManageTask(?p,?m)^ hasRiskAssessment(?m, ?r)^ hasGluRiskAssessment(?r,?g)^hasOverWeight(?g,true)^hasGluLevel(?g,glu_normal)-> hasGluRiskLevel(?g, "1"^^xsd:integer) | Guidelines for the prevention and control of type 2 diabetes in China  Page:296 paragraph:4.2 |
| D211 | SBP greater than 160 | PatientProfile(?p)^hasManageTask(?p,?m)^hasRiskAssessment(?m, ?r)^hasBPAssessment(?r,?bg)^hasSBP(?bg,?sbp)^swrlb:greaterThanOrEqual(?sbp,"160"^^xsd:integer)->hasBpRiskLevel(?bg, "3"^^xsd:integer) | Guidelines for the prevention and control of type 2 diabetes in China  Page:311 paragraph:13.2 |
| D212 | DBP greater than 110 | PatientProfile(?p)^hasManageTask(?p,?m)^hasRiskAssessment(?m, ?r)^hasBPAssessment(?r,?bg)^hasDBP(?bg,?dbp)^swrlb:greaterThanOrEqual(?dbp,"110"^^xsd:integer)->hasBpRiskLevel(?bg, "3"^^xsd:integer) | Guidelines for the prevention and control of type 2 diabetes in China  Page:311 paragraph:13.2 |
| D213 | SBP greater than 140 | PatientProfile(?p)^hasManageTask(?p,?m)^hasRiskAssessment(?m, ?r)^hasBPAssessment(?r,?bg)^hasSBP(?bg,?sbp)^swrlb:greaterThanOrEqual(?sbp,"140"^^xsd:integer)^swrlb:lessThan(?sbp,"160"^^xsd:integer)-> hasBpRiskLevel(?bg,"2"^^xsd:integer) | Guidelines for the prevention and control of type 2 diabetes in China  Page:311 paragraph:13.2 |
| D214 | DBP greater than 90 | PatientProfile(?p)^hasManageTask(?p,?m)^hasRiskAssessment(?m, ?r)^hasBPAssessment(?r,?bg)^hasDBP(?bg,?dbp)^swrlb:greaterThanOrEqual(?dbp,"90"^^xsd:integer)^swrlb:lessThan(?dbp, "110"^^xsd:integer)-> hasBpRiskLevel(?bg,"2"^^xsd:integer) | Guidelines for the prevention and control of type 2 diabetes in China  Page:311 paragraph:13.2 |
| D215 | DBP less than 140 | PatientProfile(?p)^hasManageTask(?p,?m)^hasRiskAssessment(?m, ?r)^hasBPAssessment(?r,?bg)^hasDBP(?bg,?dbp)^swrlb:lessThan(?dbp,"90"^^xsd:integer)-> hasBpRiskLevel(?bg, "1"^^xsd:integer) | Guidelines for the prevention and control of type 2 diabetes in China  Page:311 paragraph:13.2 |
| D216 | SBP less than 140 | PatientProfile(?p)^hasManageTask(?p,?m)^hasRiskAssessment(?m, ?r)^hasBPAssessment(?r,?bg)^hasSBP(?bg,?sbp)^swrlb:lessThan(?sbp,"140"^^xsd:integer)-> hasBpRiskLevel(?bg, "1"^^xsd:integer) | Guidelines for the prevention and control of type 2 diabetes in China  Page:311 paragraph:13.2 |
| D217 | total cholesterol abnormal | PatientProfile(?p)^hasManageTask(?p,?m)^hasRiskAssessment(?m,?r)^hasBFRiskAssessment(?r,?bf)^hasTC(?bf,?tc)^swrlb:greaterThanOrEqual(?tc,"3.1"^^xsd:float)^swrlb:lessThanl(?tc,"7.2"^^xsd:float)->hasBfRiskLevel(?bf, "3"^^xsd:integer) | National guidelines for the prevention and control of diabetes in primary care  Page:891 table 7 |
| D218 | LDL abnormal | PatientProfile(?p)^hasManageTask(?p,?m)^hasRiskAssessment(?m,?r)^hasBFRiskAssessment(?r,?bf)^hasLDL(?bf,?ldl)^swrlb:greaterThanOrEqual(?ldl,"1.8"^^xsd:float)^swrlb:lessThanl(?ldl,"4.9"^^xsd:float)->hasBfRiskLevel(?bf, "3"^^xsd:integer) | National guidelines for the prevention and control of diabetes in primary care  Page:891 table 7 |
| D219 | 3 risk factors | PatientProfile(?p)^hasDemographic(?p,?d)^hasGender(?d,?g)^swrlb:equal(?g,""^^xsd:string)^hasAge(?d,?a)^swrlb:greaterThanOrEqual(?a,"45"^^xsd:integer)^hasManageTask(?p,?m)^hasRiskAssessment(?m, ?r)^hasBFRiskAssessment(?r,?bf)^hasSmoking(?bf,true)->hasBfRiskLevel(?bf, "3"^^xsd:integer) | Both Clinical expert experience and medical guidelines |
| D220 | 2 risk factors | PatientProfile(?p)^hasManageTask(?p,?m)^hasRiskAssessment(?m, ?r)^hasBFRiskAssessment(?r,?bf)^hasBfRiskFactors(?bf,?rf)^swrlb:equal(?rf,"2"^^xsd:integer)-> hasBfRiskLevel(?bf, "2"^^xsd:integer) | Both Clinical expert experience and medical guidelines |
| D221 | 1 risk factor | PatientProfile(?p)^hasManageTask(?p,?m)^hasRiskAssessment(?m, ?r)^hasBFRiskAssessment(?r,?bf)^hasBfRiskFactors(?bf,?rf)^swrlb:lessThanOrEqual(?rf,"1"^^xsd:integer)->hasBfRiskLevel(?bf, "1"^^xsd:integer) | Both Clinical expert experience and medical guidelines |
| D222 | GPT less than twice | PatientProfile(?p)^hasManageTask(?p,?m)^hasRiskAssessment(?m, ?r)^hasLiverRiskAssessment(?r,?bf)^hasAlanine(?bf,?a)^swrlb:lessThan(?a,"2"^^xsd:float)-> hasLiverRiskLevel(?bf, "1"^^xsd:integer) | Both Clinical expert experience and medical guidelines |
| D223 | GOT less than twice | PatientProfile(?p)^hasManageTask(?p,?m)^hasRiskAssessment(?m, ?r)^hasLiverRiskAssessment(?r,?bf)^hasRiceStraw(?bf,?a)^swrlb:lessThan(?a,"2"^^xsd:float)-> hasLiverRiskLevel(?bf, "1"^^xsd:integer) | Both Clinical expert experience and medical guidelines |
| D224 | TBil less than twice | PatientProfile(?p)^hasManageTask(?p,?m)^hasRiskAssessment(?m, ?r)^hasLiverRiskAssessment(?r,?bf)^hasTotalBilirubin(?bf,?a)^swrlb:lessThan(?a,"2"^^xsd:float)-> hasLiverRiskLevel(?bf, "1"^^xsd:integer) | Both Clinical expert experience and medical guidelines |
| D225 | DBil less than twice | PatientProfile(?p)^hasManageTask(?p,?m)^hasRiskAssessment(?m, ?r)^hasLiverRiskAssessment(?r,?bf)^hasDirectBilirubin(?bf,?a)^swrlb:lessThan(?a,"2"^^xsd:float)->hasLiverRiskLevel(?bf, "1"^^xsd:integer) | Both Clinical expert experience and medical guidelines |
| D226 | γ-GT less than twice | PatientProfile(?p)^hasManageTask(?p,?m)^hasRiskAssessment(?m, ?r)^hasLiverRiskAssessment(?r,?bf)^hasTranspeptidase(?bf,?a)^swrlb:lessThan(?a,"2"^^xsd:float)->hasLiverRiskLevel(?bf, "1"^^xsd:integer) | Both Clinical expert experience and medical guidelines |
| D227 | GPT less than twice | PatientProfile(?p)^hasManageTask(?p,?m)^hasRiskAssessment(?m, ?r)^hasLiverRiskAssessment(?r,?bf)^hasAlanine(?bf,?a)^swrlb:lessThan(?a,"3"^^xsd:float)^swrlb:greaterThanOrEqual(?a,"2"^^xsd:float)-> hasLiverRiskLevel(?bf, "2"^^xsd:integer) | Both Clinical expert experience and medical guidelines |
| D228 | GOT less than twice | PatientProfile(?p)^hasManageTask(?p,?m)^hasRiskAssessment(?m, ?r)^hasLiverRiskAssessment(?r,?bf)^hasRiceStraw(?bf,?a)^swrlb:lessThan(?a,"3"^^xsd:float)^swrlb:greaterThanOrEqual(?a,"2"^^xsd:float)-> hasLiverRiskLevel(?bf, "2"^^xsd:integer) | Both Clinical expert experience and medical guidelines |
| D229 | TBil less than twice | PatientProfile(?p)^hasManageTask(?p,?m)^hasRiskAssessment(?m, ?r)^hasLiverRiskAssessment(?r,?bf)^hasTotalBilirubin(?bf,?a)^swrlb:lessThan(?a,"3"^^xsd:float)^swrlb:greaterThanOrEqual(?a,"2"^^xsd:float)-> hasLiverRiskLevel(?bf,"2"^^xsd:integer) | Both Clinical expert experience and medical guidelines |
| D230 | DBil less than twice | PatientProfile(?p)^hasManageTask(?p,?m)^hasRiskAssessment(?m, ?r)^hasLiverRiskAssessment(?r,?bf)^hasDirectBilirubin(?bf,?a)^swrlb:lessThan(?a,"3"^^xsd:float)^swrlb:greaterThanOrEqual(?a,"2"^^xsd:float)->hasLiverRiskLevel(?bf, "2"^^xsd:integer) | Both Clinical expert experience and medical guidelines |
| D231 | γ-GT less than twice | PatientProfile(?p)^hasManageTask(?p,?m)^hasRiskAssessment(?m,?r)^hasLiverRiskAssessment(?r,?bf)^hasTranspeptidase(?bf,?a)^^swrlb:lessThan(?a,"3"^^xsd:float)^swrlb:greaterThanOrEqual(?a,"2"^^xsd:float)-> hasLiverRiskLevel(?bf, "2"^^xsd:integer) | Both Clinical expert experience and medical guidelines |
| D232 | GPT more than twice | PatientProfile(?p)^hasManageTask(?p,?m)^hasRiskAssessment(?m, ?r)^hasLiverRiskAssessment(?r,?bf)^hasAlanine(?bf,?a)^swrlb:greaterThanOrEqual(?a,"3"^^xsd:float)->hasLiverRiskLevel(?bf, "3"^^xsd:integer) | Both Clinical expert experience and medical guidelines |
| D233 | GOT more than twice | PatientProfile(?p)^hasManageTask(?p,?m)^hasRiskAssessment(?m, ?r)^hasLiverRiskAssessment(?r,?bf)^hasRiceStraw(?bf,?a)^swrlb:greaterThanOrEqual(?a,"3"^^xsd:float)->hasLiverRiskLevel(?bf, "3"^^xsd:integer) | Both Clinical expert experience and medical guidelines |
| D234 | TBil more than twice | PatientProfile(?p)^hasManageTask(?p,?m)^hasRiskAssessment(?m, ?r)^hasLiverRiskAssessment(?r,?bf)^hasTotalBilirubin(?bf,?a)^swrlb:greaterThanOrEqual(?a,"3"^^xsd:float)->hasLiverRiskLevel(?bf, "3"^^xsd:integer) | Both Clinical expert experience and medical guidelines |
| D235 | DBil more than twice | PatientProfile(?p)^hasManageTask(?p,?m)^hasRiskAssessment(?m, ?r)^hasLiverRiskAssessment(?r,?bf)^hasDirectBilirubin(?bf,?a)^swrlb:greaterThanOrEqual(?a,"3"^^xsd:float)->hasLiverRiskLevel(?bf,"3"^^xsd:integer) | Both Clinical expert experience and medical guidelines |
| D236 | γ-GT more than twice | PatientProfile(?p)^hasManageTask(?p,?m)^hasRiskAssessment(?m, ?r)^hasLiverRiskAssessment(?r,?bf)^hasTranspeptidase(?bf,?a)^swrlb:greaterThanOrEqual(?a,"3"^^xsd:float)->hasLiverRiskLevel(?bf,"3"^^xsd:integer) | Both Clinical expert experience and medical guidelines |
| D237 | liver function  severe impairment | PatientProfile(?p)^hasManageTask(?p,?m)^hasRiskAssessment(?m, ?r)^hasLiverRiskAssessment(?r,?l)^hasLiverRiskLevel(?l,?lr)^swrlb:equal(?l,"3"^^xsd:integer)->hasRiskLevel(?r,"3"^^xsd:integer) | Clinical expert experience |
| D238 | Blood fat high risk | PatientProfile(?p)^hasManageTask(?p,?m)^hasRiskAssessment(?m, ?r)^hasBFRiskAssessment(?r,?l)^hasBfRiskLevel(?l,?bl)^swrlb:equal(?bl,"3"^^xsd:integer)->hasRiskLevel(?r,"3"^^xsd:integer) | Both Clinical expert experience and medical guidelines |
| D239 | blood pressure high risk | PatientProfile(?p)^hasManageTask(?p,?m)^hasRiskAssessment(?m, ?r)^hasBPRiskAssessment(?r,?l)^hasBpRiskLevel(?l,?bp)^swrlb:equal(?bp,"3"^^xsd:integer)->hasRiskLevel(?r,"3"^^xsd:integer) | Both Clinical expert experience and medical guidelines |
| D240 | blood glucosehigh risk | PatientProfile(?p)^hasManageTask(?p,?m)^hasRiskAssessment(?m, ?r)^hasGluRiskAssessment(?r,?l)^hasGluRiskLevel(?l,?glu)^swrlb:equal(?glu,"3"^^xsd:integer)->hasRiskLevel(?r,"3"^^xsd:integer) | Both Clinical expert experience and medical guidelines |
| D241 | liver function  moderate impairment with intermediate-risk blood fat | PatientProfile(?p)^hasManageTask(?p,?m)^ hasRiskAssessment(?m, ?r)^hasLiverRiskAssessment(?r,?l)^hasLiverRiskLevel(?l,?lr)^swrlb:equal(?l,"2"^^xsd:integer)^hasBFRiskAssessment(?r,?l)^hasBfRiskLevel(?l,?bl)^swrlb:equal(?bl,"2"^^xsd:integer)->hasRiskLevel(?r,"2"^^xsd:integer) | Clinical expert experience |
| D242 | liver function  moderate impairment with intermediate-risk blood pressure | PatientProfile(?p)^hasManageTask(?p,?m)^ hasRiskAssessment(?m, ?r)^hasLiverRiskAssessment(?r,?l)^hasLiverRiskLevel(?l,?lr)^swrlb:equal(?l,"2"^^xsd:integer)^hasBPRiskAssessment(?r,?l)^hasBpRiskLevel(?l,?bp)^swrlb:equal(?bp,"2"^^xsd:integer)->hasRiskLevel(?r,"2"^^xsd:integer) | Clinical expert experience |
| D243 | liver function  moderate impairment with intermediate-risk blood glucose | PatientProfile(?p)^hasManageTask(?p,?m)^hasRiskAssessment(?m, ?r)^hasLiverRiskAssessment(?r,?l)^hasLiverRiskLevel(?l,?lr)^swrlb:equal(?l,"2"^^xsd:integer)^hasGluRiskAssessment(?r,?l)^hasGluRiskLevel(?l,?glu)^swrlb:equal(?glu,"2"^^xsd:integer)->hasRiskLevel(?r,"2"^^xsd:integer) | Clinical expert experience |
| D244 | intermediate-risk blood fat with intermediate-risk blood pressure | PatientProfile(?p)^hasManageTask(?p,?m)^ hasRiskAssessment(?m, ?r)^hasBFRiskAssessment(?r,?l)^hasBfRiskLevel(?l,?bl)^swrlb:equal(?bl,"2"^^xsd:integer)^hasBPRiskAssessment(?r,?l)^hasBpRiskLevel(?l,?bp)^swrlb:equal(?bp,"2"^^xsd:integer)->hasRiskLevel(?r,"2"^^xsd:integer) | Both Clinical expert experience and medical guidelines |
| D245 | ntermediate-risk blood fat with intermediate-risk blood glucose | PatientProfile(?p)^hasManageTask(?p,?m)^ hasRiskAssessment(?m, ?r)^hasBFRiskAssessment(?r,?l)^hasBfRiskLevel(?l,?bl)^swrlb:equal(?bl,"2"^^xsd:integer)^hasGluRiskAssessment(?r,?l)^hasGluRiskLevel(?l,?glu)^swrlb:equal(?glu,"2"^^xsd:integer)->hasRiskLevel(?r,"2"^^xsd:integer) | Both Clinical expert experience and medical guidelines |
| D246 | intermediate-risk blood pressure with intermediate-risk blood glucose | PatientProfile(?p)^hasManageTask(?p,?m)^ hasRiskAssessment(?m, ?r)^hasBPRiskAssessment(?r,?l)^hasBpRiskLevel(?l,?bp)^swrlb:equal(?bp,"2"^^xsd:integer)^hasGluRiskAssessment(?r,?l)^hasGluRiskLevel(?l,?glu)^swrlb:equal(?glu,"2"^^xsd:integer)->hasRiskLevel(?r,"2"^^xsd:integer) | Both Clinical expert experience and medical guidelines |
| D247 | liver function mild impairment with bp and bf low-risk | PatientProfile(?p)^hasManageTask(?p,?m)^hasRiskAssessment(?m, ?r)^hasLiverRiskAssessment(?r,?l)^hasLiverRiskLevel(?l,?lr)^swrlb:equal(?l,"1"^^xsd:integer)^hasBFRiskAssessment(?r,?l)^hasBfRiskLevel(?l,?bl)^swrlb:equal(?bl,"1"^^xsd:integer)^hasBPRiskAssessment(?r,?l)^hasBpRiskLevel(?l,?bp)^swrlb:equal(?bp,"1"^^xsd:integer)->hasRiskLevel(?r,"1"^^xsd:integer) | Clinical expert experience |
| D248 | liver function mild impairment with bf and bg low-risk | PatientProfile(?p)^hasManageTask(?p,?m)^ hasRiskAssessment(?m, ?r)^hasLiverRiskAssessment(?r,?l)^hasLiverRiskLevel(?l,?lr)^swrlb:equal(?l,"1"^^xsd:integer)^hasBFRiskAssessment(?r,?l)^hasBfRiskLevel(?l,?bl)^swrlb:equal(?bl,"1"^^xsd:integer)^hasGluRiskAssessment(?r,?l)^hasGluRiskLevel(?l,?glu)^swrlb:equal(?glu,"1"^^xsd:integer)->hasRiskLevel(?r,"1"^^xsd:integer) | Clinical expert experience |
| D249 | liver function mild impairment with bp and bg low-risk | PatientProfile(?p)^hasManageTask(?p,?m)^hasRiskAssessment(?m, ?r)^hasLiverRiskAssessment(?r,?l)^hasLiverRiskLevel(?l,?lr)^swrlb:equal(?l,"1"^^xsd:integer)^hasBPRiskAssessment(?r,?l)^hasBpRiskLevel(?l,?bp)^swrlb:equal(?bp,"1"^^xsd:integer)^hasGluRiskAssessment(?r,?l)^hasGluRiskLevel(?l,?glu)^swrlb:equal(?glu,"1"^^xsd:integer)->hasRiskLevel(?r,"1"^^xsd:integer) | Clinical expert experience |
| D250 | bf ,bg and bf low-risk | PatientProfile(?p)^hasManageTask(?p,?m)^hasRiskAssessment(?m, ?r)^hasBFRiskAssessment(?r,?l)^hasBfRiskLevel(?l,?bl)^swrlb:equal(?bl,"1"^^xsd:integer)^hasBPRiskAssessment(?r,?l)^hasBpRiskLevel(?l,?bp)^swrlb:equal(?bp,"1"^^xsd:integer)^hasGluRiskAssessment(?r,?l)^hasGluRiskLevel(?l,?glu)^swrlb:equal(?glu,"1"^^xsd:integer)->hasRiskLevel(?r,"1"^^xsd:integer) | Clinical expert experience |
| D251 | liver function mild impairment with bp,bf and bg low-risk | PatientProfile(?p)^hasManageTask(?p,?m)^hasRiskAssessment(?m, ?r)^hasBFRiskAssessment(?r,?l)^hasBfRiskLevel(?l,?bl)^swrlb:equal(?bl,"1"^^xsd:integer)^hasBPRiskAssessment(?r,?l)^hasBpRiskLevel(?l,?bp)^swrlb:equal(?bp,"1"^^xsd:integer)^hasGluRiskAssessment(?r,?l)^hasGluRiskLevel(?l,?glu)^swrlb:equal(?glu,"1"^^xsd:integer)^hasLiverRiskAssessment(?r,?l)^hasLiverRiskLevel(?l,?lr)^swrlb:equal(?l,"1"^^xsd:integer)->hasRiskLevel(?r,"1"^^xsd:integer) | Clinical expert experience |

Table 3 Expression results of SWRL rules for diabetes comprehensive control targets

| serial | | rules | | rules definition | | source | |
| --- | --- | --- | --- | --- | --- | --- | --- |
| D301 | FBG | | PatientProfile(?p)^hasDiabetesDiagnosis(?p,true) ^hasManageTask(?p,?m)^hasHierarchicalManagement(?m,?h)^ hasManagementLevel(?h, ?l)^hasMonitoringSubplan(?h,?ms) -> hasManageItem(?ms, GLUMeasure) | | Guidelines for the prevention and control of type 2 diabetes in China  Page:301 table 7 | |  |
| D302 | PBG | | PatientProfile(?p)^hasDiabetesDiagnosis(?p,true) ^hasManageTask(?p,?m)^hasHierarchicalManagement(?m,?h)^ hasManagementLevel(?h,?l)^hasMonitoringSubplan(?h,?ms) -> hasManageItem(?ms, PBGMeasure) | | Guidelines for the prevention and control of type 2 diabetes in China  Page:301 table 7 | |  |
| D303 | BMI | | PatientProfile(?p)^hasDiabetesDiagnosis(?p,true) ^hasManageTask(?p,?m)^hasHierarchicalManagement(?m,?h)^ hasManagementLevel(?h, ?l)^hasMonitoringSubplan(?h,?ms) -> hasManageItem(?ms, WeightMeasure) | | Guidelines for the prevention and control of type 2 diabetes in China  Page:301 table 7 | |  |
| D304 | SBP | | PatientProfile(?p)^hasDiabetesDiagnosis(?p,true) ^hasManageTask(?p,?m)^hasHierarchicalManagement(?m,?h)^ hasManagementLevel(?h, ?l)^hasMonitoringSubplan(?h,?ms) -> hasManageItem(?ms, SbpMeasure) | | Guidelines for the prevention and control of type 2 diabetes in China  Page:301 table 7 | |  |
| D305 | DBP | | PatientProfile(?p)^hasDiabetesDiagnosis(?p,true) ^hasManageTask(?p,?m)^hasHierarchicalManagement(?m,?h)^ hasManagementLevel(?h, ?l)^hasMonitoringSubplan(?h,?ms) -> hasManageItem(?ms, DbpMeasure) | | Guidelines for the prevention and control of type 2 diabetes in China  Page:301 table 7 | |  |
| D306 | DF | | PatientProfile(?p)^hasDiabetesDiagnosis(?p,true) ^hasManageTask(?p,?m)^hasHierarchicalManagement(?m,?h)^ hasManagementLevel(?h, ?l)^hasMonitoringSubplan(?h,?ms) -> hasManageItem(?ms, LDLCMeasure) | | Guidelines for the prevention and control of type 2 diabetes in China  Page:301 table 7 | |  |
| D307 | loose control target | | PatientProfile(?p)^hasDiabetesDiagnosis(?p,true)^hasManageTask(?p,?m)^hasHierarchicalManagement(?m,?h)^ hasRiskAssessment(?m, ?r) ^hasRiskLevel(?r,?l)^hasMonitoringSubplan(?h,?ms)^hasManageItem(?ms,GLUMeasure)^hasManageItem(?ms,PBGMeasure)^ hasManageItem(?ms,WeightMeasure)^swrlb:equal(?l,"1"^^xsd:integer)->hasControlTarget(GLUMeasure,"7.8-10.0")^ hasControlTarget  (PBGMeasure,"7.8-13.9")^hasControlTarget(WeightMeasure, "BMI<24") | | Both Clinical expert experience and medical guidelines | |  |
| D308 | common control target | | PatientProfile(?p)^hasDiabetesDiagnosis(?p,true)^hasManageTask(?p,?m)^hasHierarchicalManagement(?m,?h)^ hasRiskAssessment(?m, ?r) ^hasRiskLevel(?r,?l)^hasMonitoringSubplan(?h,?ms)^hasManageItem(?ms,GLUMeasure)^hasManageItem(?ms,PBGMeasure)^hasManageItem(?ms,WeightMeasure)^swrlb:equal(?l,"2"^^xsd:integer)-> hasControlTarget(GLUMeasure,"6.1-7.8")^ hasControlTarget  (PBGMeasure,"7.8-10.0")^hasControlTarget(WeightMeasure, "BMI<24") | | Both Clinical expert experience and medical guidelines | |  |
| D309 | Strictly control target | | PatientProfile(?p)^hasDiabetesDiagnosis(?p,true)^hasManageTask(?p,?m)^hasHierarchicalManagement(?m,?h)^hasRiskAssessment(?m,?r)^hasRiskLevel(?r, ?l)^hasMonitoringSubplan(?h,?ms)^hasManageItem(?ms,GLUMeasure)^hasManageItem(?ms,PBGMeasure)^hasManageItem(?ms,WeightMeasure)^swrlb:equal(?l,"3"^^xsd:integer)->hasControlTarget(GLUMeasure,"4.4-6.1")^hasControlTarget(PBGMeasure, "6.1-7.8") ^ hasControlTarget(WeightMeasure, "BMI<24") | | Both Clinical expert experience and medical guidelines | |  |

Table 4 Expression results of SWRL rules for diabetes hierarchical management

| serial | rules | rules definition | source |
| --- | --- | --- | --- |
| D401 | General management(standard-1) | PatientProfile(?p)^hasDiabetesDiagnosis(?p,true)^ hasManageTask(?p,?m)^hasHierarchicalManagement(?m, ?h) ^hasRiskAssessment(?m,?r)^hasRiskLevel  (?r,?v)^swrlb:equal(?v,"3"^^xsd:integer)^hasMonthlyGlu(?h,?glu)^hasMonthlyPbg(?h,?pbg)^swrlb:lessThanOrEqual(?glu, "6.1"^^xsd:float)^swrlb:greaterThanOrEqual(?glu, "4.4"^^xsd:float)^swrlb:lessThanOrEqual(?pbg, "7.8"^^xsd:float)^swrlb:greaterThanOrEqual(?pbg, "6.1"^^xsd:float)->hasManagementLevel(?h,"1"^^xsd:integer) | Clinical expert experience |
| D402 | General management(standard-2) | PatientProfile(?p) ^ hasDiabetesDiagnosis(?p, true) ^ hasManageTask(?p,?m)^hasHierarchicalManagement(?m, ?h) ^ hasRiskAssessment(?m, ?r) ^ hasRiskLevel(?r, ?v) ^ swrlb:equal(?v, "2"^^xsd:integer) ^ hasMonthlyGlu(?h, ?glu) ^ hasMonthlyPbg(?h, ?pbg) ^ swrlb:lessThanOrEqual(?glu, "7.8"^^xsd:float)^swrlb:greaterThanOrEqual(?glu, "6.1"^^xsd:float)^swrlb:lessThanOrEqual(?pbg, "10.0"^^xsd:float)^swrlb:greaterThanOrEqual(?pbg, "7.8"^^xsd:float)->hasManagementLevel(?h,"1"^^xsd:integer) | Clinical expert experience |
| D403 | General management(standard-3) | PatientProfile(?p)^hasDiabetesDiagnosis(?p,true) ^ hasManageTask(?p,?m)^hasHierarchicalManagement(?m, ?h) ^ hasRiskAssessment(?m,?r)^hasRiskLevel(?r,?v)^ swrlb:equal(?v,"1"^^xsd:integer)^hasMonthlyGlu(?h,?glu)^ hasMonthlyPbg(?h,?pbg)^swrlb:lessThanOrEqual(?glu, "10.0"^^xsd:float)^swrlb:greaterThanOrEqual(?glu, "7.8"^^xsd:float)^swrlb:lessThanOrEqual(?pbg, "13.9"^^xsd:float)^swrlb:greaterThanOrEqual(?pbg, "10.0"^^xsd:float)->hasManagementLevel(?h,"1"^^xsd:integer) | Clinical expert experience |
| D404 | General management(substandard-1) | PatientProfile(?p) ^ hasDiabetesDiagnosis(?p, true) ^ hasManageTask(?p,?m)^hasHierarchicalManagement(?m, ?h) ^ hasRiskAssessment(?m, ?r) ^ hasRiskLevel(?r, ?v) ^ swrlb:equal(?v,"3"^^xsd:integer)^ hasMonthlyGlu  (?h,?glu)^hasMonthlyPbg(?h,?pbg)^ swrlb:lessThan  (?glu,"4.4"^^xsd:float)^ swrlb:lessThan(?pbg,  "16.7"^^xsd:float)->hasManagementLevel(?h, "2"^^xsd:integer) | Clinical expert experience |
| D405 | General management(substandard-2) | PatientProfile(?p) ^ hasDiabetesDiagnosis(?p, true) ^ hasManageTask(?p,?m)^hasHierarchicalManagement(?m, ?h) ^ hasRiskAssessment(?m, ?r) ^ hasRiskLevel(?r, ?v) ^ swrlb:equal(?v,"3"^^xsd:integer)^ hasMonthlyGlu  (?h,?glu)^hasMonthlyPbg(?h,?pbg)^ swrlb:lessThan  (?glu,"11.1"^^xsd:float)^swrlb:greaterThan(?glu,"6.1"^^xsd:float)^swrlb:lessThan(?pbg,"16.7"^^xsd:float)-> hasManagementLevel(?h, "2"^^xsd:integer) | Clinical expert experience |
| D406 | General management(substandard-3) | PatientProfile(?p) ^ hasDiabetesDiagnosis(?p, true) ^ hasManageTask(?p,?m)^hasHierarchicalManagement(?m, ?h) ^ hasRiskAssessment(?m, ?r) ^ hasRiskLevel(?r, ?v) ^ swrlb:equal(?v,"3"^^xsd:integer)^ hasMonthlyGlu  (?h,?glu)^hasMonthlyPbg(?h,?pbg)^ swrlb:lessThan  (?glu,"11.1"^^xsd:float)^ swrlb:lessThan  (?pbg,"6.1"^^xsd:float)->hasManagementLevel(?h, "2"^^xsd:integer) | Clinical expert experience |
| D407 | General management(substandard-4) | PatientProfile(?p) ^ hasDiabetesDiagnosis(?p, true) ^ hasManageTask(?p,?m)^hasHierarchicalManagement(?m, ?h) ^ hasRiskAssessment(?m, ?r) ^ hasRiskLevel(?r, ?v) ^ swrlb:equal(?v,"3"^^xsd:integer)^ hasMonthlyGlu  (?h,?glu)^hasMonthlyPbg(?h,?pbg)^ swrlb:lessThan  (?glu,"11.1"^^xsd:float)^ swrlb:greaterThan  (?pbg,"7.8"^^xsd:float)^ swrlb:lessThan  (?pbg"16.7"^^xsd:float)->hasManagementLevel(?h, "2"^^xsd:integer) | Clinical expert experience |
| D408 | General management(substandard-5) | PatientProfile(?p) ^ hasDiabetesDiagnosis(?p, true) ^ hasManageTask(?p,?m)^hasHierarchicalManagement(?m, ?h) ^ hasRiskAssessment(?m, ?r) ^ hasRiskLevel(?r, ?v) ^ swrlb:equal(?v, "2"^^xsd:integer) ^ hasMonthlyGlu(?h, ?glu) ^ hasMonthlyPbg(?h, ?pbg) ^ swrlb:lessThan(?glu, "6.1"^^xsd:float) ^ swrlb:lessThan(?pbg, "16.7"^^xsd:float) -> hasManagementLevel(?h, "2"^^xsd:integer) | Clinical expert experience |
| D409 | General management(substandard-6) | PatientProfile(?p) ^ hasDiabetesDiagnosis(?p, true) ^ hasManageTask(?p,?m)^hasHierarchicalManagement(?m, ?h) ^ hasRiskAssessment(?m, ?r) ^ hasRiskLevel(?r, ?v) ^ swrlb:equal(?v,"2"^^xsd:integer)^ hasMonthlyGlu(?h, ?glu) ^ hasMonthlyPbg(?h, ?pbg) ^ swrlb:lessThan(?glu,"11.1"^^xsd:float) ^swrlb:greaterThan(?glu,"7.8"^^xsd:float)^ swrlb:lessThan(?pbg,"16.7"^^xsd:float)-> hasManagementLevel(?h, "2"^^xsd:integer) | Clinical expert experience |
| D410 | General management(substandard-7) | atientProfile(?p) ^ hasDiabetesDiagnosis(?p, true) ^ hasManageTask(?p,?m)^hasHierarchicalManagement(?m, ?h) ^ hasRiskAssessment(?m, ?r) ^ hasRiskLevel(?r, ?v) ^ swrlb:equal(?v,"2"^^xsd:integer)^ hasMonthlyGlu(?h, ?glu) ^ hasMonthlyPbg(?h, ?pbg) ^ swrlb:lessThan(?glu,"11.1"^^xsd:float)^ swrlb:lessThan(?pbg,"7.8"^^xsd:float)-> hasManagementLevel(?h, "2"^^xsd:integer) | Clinical expert experience |
| D411 | General management(substandard-8) | PatientProfile(?p) ^ hasDiabetesDiagnosis(?p, true) ^ hasManageTask(?p,?m)^hasHierarchicalManagement(?m, ?h) ^ hasRiskAssessment(?m, ?r) ^ hasRiskLevel(?r, ?v) ^ swrlb:equal(?v, "2"^^xsd:integer) ^ hasMonthlyGlu(?h, ?glu) ^ hasMonthlyPbg(?h, ?pbg) ^ swrlb:lessThan(?glu, "11.1"^^xsd:float) ^ swrlb:greaterThan(?pbg, "10.0"^^xsd:float)^ swrlb:lessThan(?pbg, "16.7"^^xsd:float) -> hasManagementLevel(?h, "2"^^xsd:integer) | Clinical expert experience |
| D412 | General management(substandard-9) | PatientProfile(?p) ^ hasDiabetesDiagnosis(?p, true) ^ hasManageTask(?p,?m)^hasHierarchicalManagement(?m, ?h) ^ hasRiskAssessment(?m, ?r) ^ hasRiskLevel(?r, ?v) ^ swrlb:equal(?v, "1"^^xsd:integer) ^ hasMonthlyGlu(?h, ?glu) ^ hasMonthlyPbg(?h, ?pbg) ^ swrlb:lessThan(?glu, "7.8"^^xsd:float) ^ swrlb:lessThan(?pbg, "16.7"^^xsd:float) -> hasManagementLevel(?h, "2"^^xsd:integer) | Clinical expert experience |
| D413 | General management(substandard-10) | PatientProfile(?p) ^ hasDiabetesDiagnosis(?p, true) ^ hasManageTask(?p,?m)^hasHierarchicalManagement(?m, ?h) ^ hasRiskAssessment(?m, ?r) ^ hasRiskLevel(?r, ?v) ^ swrlb:equal(?v,"1"^^xsd:integer) ^ hasMonthlyGlu(?h, ?glu) ^ hasMonthlyPbg(?h, ?pbg) ^ swrlb:lessThan(?glu,"11.1"^^xsd:float) ^swrlb:greaterThan(?glu,"10.0"^^xsd:float)^ swrlb:lessThan(?pbg, "16.7"^^xsd:float) -> hasManagementLevel(?h, "2"^^xsd:integer) | Clinical expert experience |
| D414 | Strengthen management-1 | PatientProfile(?p) ^ hasDiabetesDiagnosis(?p, true) ^ hasManageTask(?p,?m)^hasHierarchicalManagement(?m, ?h) ^ hasRiskAssessment(?m, ?r) ^ hasRiskLevel(?r, ?v) ^ swrlb:equal(?v, "1"^^xsd:integer) ^ hasMonthlyGlu(?h, ?glu) ^ hasMonthlyPbg(?h, ?pbg) ^ swrlb:lessThan(?glu, "11.1"^^xsd:float) ^ swrlb:lessThan(?pbg, "7.8"^^xsd:float) -> hasManagementLevel(?h, "3"^^xsd:integer) | Clinical expert experience |
| D415 | Strengthen management-2 | PatientProfile(?p) ^ hasDiabetesDiagnosis(?p, true) ^ hasManageTask(?p,?m)^hasHierarchicalManagement(?m, ?h) ^ hasRiskAssessment(?m, ?r) ^ hasRiskLevel(?r, ?v) ^ swrlb:equal(?v, "1"^^xsd:integer) ^ hasMonthlyGlu(?h, ?glu) ^ hasMonthlyPbg(?h, ?pbg) ^ swrlb:lessThan(?glu, "11.1"^^xsd:float) ^ swrlb:greaterThan(?pbg,"13.9"^^xsd:float)^ swrlb:lessThan(?pbg, "16.7"^^xsd:float) -> hasManagementLevel(?h, "3"^^xsd:integer) | Clinical expert experience |
| D416 | Strengthen management-3 | PatientProfile(?p) ^ hasDiabetesDiagnosis(?p, true) ^ hasManageTask(?p,?m)^hasHierarchicalManagement(?m, ?h) ^ hasRiskAssessment(?m, ?r) ^ hasRiskLevel(?r, ?v) ^ swrlb:lessThanOrEqual(?v,"3"^^xsd:integer)^ hasMonthlyGlu(?h,?glu)^ swrlb:greaterThanOrEqual(?glu, "11.1"^^xsd:float)->hasManagementLevel(?h, "3"^^xsd:integer) | Clinical expert experience |
| D417 | Strengthen management-4 | PatientProfile(?p) ^ hasDiabetesDiagnosis(?p, true) ^ hasManageTask(?p,?m)^hasHierarchicalManagement(?m, ?h) ^ hasRiskAssessment(?m, ?r) ^ hasRiskLevel(?r, ?v) ^ swrlb:lessThanOrEqual(?v,"3"^^xsd:integer)^ hasMonthlyPbg(?h,?pbg)^ swrlb:greaterThanOrEqual  (?pbg,"16.7"^^xsd:float)->hasManagementLevel(?h, "3"^^xsd:integer) | Clinical expert experience |
| D418 | Initialization management | PatientProfile(?p) ^ hasDiabetesDiagnosis(?p, true) ^ hasManageTask(?p,?m)^ hasHierarchicalManagement  (?p,?h)^hasInitialPeriod(?h,true)-> hasManagementLevel  (?h, 0) | Clinical expert experience |

Table 5 Expression results of SWRL rules for diabetes hierarchical management

| serial | rules | rules definition | source |
| --- | --- | --- | --- |
| D501 | single drug dosage with Management standard-1 | PatientProfile(?p)^hasDiabetesDiagnosis(?p,true) ^hasManageTask  (?p,?m)^hasHierarchicalManagement(?m,?h)^ hasManagementLevel(?h,?l)^hasMonitoringSubplan(?h,?ms)-> hasManageItem(?ms, “GLU01”) | Both Clinical expert experience and medical guidelines |
| D502 | single drug dosage with Management standard-2 | PatientProfile(?p)^hasDiabetesDiagnosis(?p,true) ^hasManageTask  (?p,?m)^hasHierarchicalManagement(?m,?h)^ hasManagementLevel(?h,?l)^hasMonitoringSubplan(?h,?ms)-> hasManageItem(?ms, “BP01”) | Both Clinical expert experience and medical guidelines |
| D503 | single drug dosage with Management substandard-1 | PatientProfile(?p)^hasDiabetesDiagnosis(?p,true) ^hasManageTask  (?p,?m)^hasHierarchicalManagement(?m,?h)^ hasManagementLevel(?h,?l)^hasMonitoringSubplan(?h,?ms)-> hasManageItem(?ms, “GLU02”) | Both Clinical expert experience and medical guidelines |
| D504 | single drug dosage with Management substandard-1 | PatientProfile(?p)^hasDiabetesDiagnosis(?p,true) ^hasManageTask  (?p,?m)^hasHierarchicalManagement(?m,?h)^ hasManagementLevel(?h,?l)^hasMonitoringSubplan(?h,?ms)-> hasManageItem(?ms, “BP02”) | Both Clinical expert experience and medical guidelines |
| D505 | double drug dosage with Management standard-1 | PatientProfile(?p)^hasDiabetesDiagnosis(?p,true) ^hasManageTask(?p,?m)^hasHierarchicalManagement(?m,?h)^ hasManagementLevel(?h,?l)^hasMonitoringSubplan(?h,?ms)-> hasManageItem(?ms, “BP03”) | Both Clinical expert experience and medical guidelines |
| D506 | double drug dosage with Management standard-2 | PatientProfile(?p)^hasDiabetesDiagnosis(?p,true) ^hasManageTask(?p,?m)^hasHierarchicalManagement(?m,?h)^ hasManagementLevel(?h,?l)^hasMonitoringSubplan(?h,?ms)-> hasManageItem(?ms, “GLU03”) | Both Clinical expert experience and medical guidelines |
| D507 | double drug dosage with Management substandard-1 | PatientProfile(?p)^hasDiabetesDiagnosis(?p,true) ^hasManageTask(?p,?m)^hasHierarchicalManagement(?m,?h)^ hasManagementLevel(?h,?l)^hasMonitoringSubplan(?h,?ms)-> hasManageItem(?ms, “GLU04”) | Both Clinical expert experience and medical guidelines |
| D508 | double drug dosage with Management substandard-2 | PatientProfile(?p)^hasDiabetesDiagnosis(?p,true) ^hasManageTask(?p,?m)^hasHierarchicalManagement(?m,?h)^ hasManagementLevel(?h,?l)^hasMonitoringSubplan(?h,?ms)-> hasManageItem(?ms, “BP04”) | Both Clinical expert experience and medical guidelines |
| D509 | triple drug dosage with Management standard-1 | PatientProfile(?p)^hasDiabetesDiagnosis(?p,true) ^hasManageTask(?p,?m)^hasHierarchicalManagement(?m,?h)^ hasManagementLevel(?h,?l)^hasMonitoringSubplan(?h,?ms)-> hasManageItem(?ms, “GLU05”) | Both Clinical expert experience and medical guidelines |
| D510 | triple drug dosage with Management standard-2 | PatientProfile(?p)^hasDiabetesDiagnosis(?p,true) ^hasManageTask(?p,?m)^hasHierarchicalManagement(?m,?h)^ hasManagementLevel(?h,?l)^hasMonitoringSubplan(?h,?ms)-> hasManageItem(?ms, “BP05”) | Both Clinical expert experience and medical guidelines |
| D511 | triple drug dosage with Management substandard-1 | PatientProfile(?p)^hasDiabetesDiagnosis(?p,true) ^hasManageTask(?p,?m)^hasHierarchicalManagement(?m,?h)^ hasManagementLevel(?h,?l)^hasMonitoringSubplan(?h,?ms)-> hasManageItem(?ms, “GLU06”) | Both Clinical expert experience and medical guidelines |
| D512 | triple drug dosage with Management substandard-1 | PatientProfile(?p)^hasDiabetesDiagnosis(?p,true) ^hasManageTask(?p,?m)^hasHierarchicalManagement(?m,?h)^ hasManagementLevel(?h,?l)^hasMonitoringSubplan(?h,?ms)-> hasManageItem(?ms, “BP06”) | Both Clinical expert experience and medical guidelines |
| D513 | insulin with Management standard-1 | PatientProfile(?p)^hasDiabetesDiagnosis(?p,true) ^hasManageTask(?p,?m)^hasHierarchicalManagement(?m,?h)^ hasManagementLevel(?h,?l)^hasMonitoringSubplan(?h,?ms)-> hasManageItem(?ms, “GLU07”) | Both Clinical expert experience and medical guidelines |
| D514 | insulin with Management standard-2 | PatientProfile(?p)^hasDiabetesDiagnosis(?p,true) ^hasManageTask(?p,?m)^hasHierarchicalManagement(?m,?h)^ hasManagementLevel(?h,?l)^hasMonitoringSubplan(?h,?ms)-> hasManageItem(?ms, “BP07”) | Both Clinical expert experience and medical guidelines |
| D515 | insulin with Management substandard-1 | PatientProfile(?p)^hasDiabetesDiagnosis(?p,true) ^hasManageTask(?p,?m)^hasHierarchicalManagement(?m,?h)^ hasManagementLevel(?h,?l)^hasMonitoringSubplan(?h,?ms)-> hasManageItem(?ms, “GLU08”) | Both Clinical expert experience and medical guidelines |
| D516 | insulin with Management substandard-2 | PatientProfile(?p)^hasDiabetesDiagnosis(?p,true) ^hasManageTask(?p,?m)^hasHierarchicalManagement(?m,?h)^ hasManagementLevel(?h,?l)^hasMonitoringSubplan(?h,?ms)-> hasManageItem(?ms, “BP08”) | Both Clinical expert experience and medical guidelines |
| D517 | insulin with Management severe substandard-1 | PatientProfile(?p)^hasDiabetesDiagnosis(?p,true) ^hasManageTask(?p,?m)^hasHierarchicalManagement(?m,?h)^ hasManagementLevel(?h,?l)^hasMonitoringSubplan(?h,?ms)-> hasManageItem(?ms, “GLU09”) | Both Clinical expert experience and medical guidelines |
| D518 | insulin with Management severe substandard-2 | PatientProfile(?p)^hasDiabetesDiagnosis(?p,true) ^hasManageTask(?p,?m)^hasHierarchicalManagement(?m,?h)^ hasManagementLevel(?h,?l)^hasMonitoringSubplan(?h,?ms)-> hasManageItem(?ms, “BP09”) | Both Clinical expert experience and medical guidelines |

Table 6 Expression results of SWRL rules for diabetes regular follow-up

| serial | rules | rules definition | source |
| --- | --- | --- | --- |
| D601 | follow-up not completed-1 | PatientProfile(?p)·^hasDiabetesDiagnosis(?p,true)^ hasManageTask(?p,?m)^hashasFollowupScheduling(?m,?f)^ hasUnfinishedFollowup(?f,true)^hasManagementLevel(?p,?l)^ swrlb:equal(?l,"1"^^xsd:integer)->hasNewFollowup(?f,"0"^^xsd:integer) | Both Clinical expert experience and medical guidelines |
| D602 | follow-up not completed-2 | PatientProfile(?p)^hasDiabetesDiagnosis(?p,true)^ hasManageTask  (?p,?m)^hasFollowupScheduling(?m,?f)^ swrlb:equal  (?l,"2"^^xsd:integer)^swrlb:greaterThan(?d, "30"^^xsd:integer)  ^hasDaysToFollowup(?f,?d)^ hasManagementLevel  (?p, ?l) ^ hasUnfinishedFollowup(?f, true) -> hasNewFollowup(?f, "30"^^xsd:integer) | Both Clinical expert experience and medical guidelines |
| D603 | follow-up not completed-3 | PatientProfile(?p)^hasDiabetesDiagnosis(?p,true)^ hasManageTask  (?p,?m)^hasFollowupScheduling(?m,?f)^ swrlb:equal  (?l"2"^^xsd:integer)^swrlb:lessThanOrEqual(?d, "30"^^xsd:integer)  ^hasDaysToFollowup(?f,?d)^hasManagementLevel(?p,?l)^ hasUnfinishedFollowup(?f,true)->hasNewFollowup(?f,"0"^^xsd:integer) | Both Clinical expert experience and medical guidelines |
| D604 | follow-up not completed-4 | PatientProfile(?p)^hasDiabetesDiagnosis(?p,true)^ hasManageTask  (?p,?m)^hasFollowupScheduling(?m,?f)^ swrlb:equal  (?l,"3"^^xsd:integer)^swrlb:greaterThan(?d, "14"^^xsd:integer)  ^hasDaysToFollowup(?f,?d)^ hasManagementLevel(?p,?l)^  hasUnfinishedFollowup(?f,true)->hasNewFollowup(?f,"14"^^xsd:integer) | Both Clinical expert experience and medical guidelines |
| D605 | follow-up not completed-5 | PatientProfile(?p)^hasDiabetesDiagnosis(?p,true)^hasManageTask(?p,?m)^hasFollowupScheduling(?m,?f)^swrlb:equal(?l, "3"^^xsd:integer) ^swrlb:lessThanOrEqual(?d,"14"^^xsd:integer)^hasDaysToFollowup(?f,?d)^hasManagementLevel(?p, ?l) ^ hasUnfinishedFollowup(?f, true) -> hasNewFollowup(?f, "0"^^xsd:integer) | Both Clinical expert experience and medical guidelines |
| D606 | follow-up completed-1 | PatientProfile(?p)^hasDiabetesDiagnosis(?p,true)^hasManageTask(?p,?m)^hasFollowupScheduling(?m,?f)^hasUnfinishedFollowup(?f,false)^hasManagementLevel(?p,?l)^swrlb:equal(?l,"1"^^xsd:integer)->hasNewFollowup(?f, "90"^^xsd:integer) | Both Clinical expert experience and medical guidelines |
| D607 | follow-up completed-2 | PatientProfile(?p)^hasDiabetesDiagnosis(?p,true)^hasManageTask(?p,?m)^hasFollowupScheduling(?m,?f)^hasUnfinishedFollowup(?f,false)^hasManagementLevel(?p,?l)^swrlb:equal(?l,"2"^^xsd:integer)-> hasNewFollowup(?f, "30"^^xsd:integer) | Both Clinical expert experience and medical guidelines |
| D608 | follow-up completed-3 | PatientProfile(?p)^hasDiabetesDiagnosis(?p,true)^hasManageTask(?p,?m)^hasFollowupScheduling(?m,?f)^hasUnfinishedFollowup(?f,false)^hasManagementLevel(?p,?l)^swrlb:equal(?l,"3"^^xsd:integer)-> hasNewFollowup(?f, "14"^^xsd:integer) | Both Clinical expert experience and medical guidelines |

Table 7 Expression results of SWRL rules for diabetes abnormal attention

| serial | rules | rules definition | source |
| --- | --- | --- | --- |
| D701 | hypoglycemia | PatientProfile(?p)^hasDiabetesDiagnosis(?p,true) ^hasManageTask(?p,?m)^hasAbnormalWarning(?m, ?a) ^ hasGlu(?p, ?g) ^ swrlb:lessThanOrEqual(?g, "3.9"^^xsd:float) -> hasWarningCode(?a, "G01") | Clinical expert experience |
| D702 | low bg | PatientProfile(?p) ^ hasDiabetesDiagnosis(?p, true) ^ hasManageTask(?p,?m)^ hasAbnormalWarning(?m, ?a)^ hasGlu(?p, ?g) ^ swrlb:lessThanOrEqual(?g, "4.4"^^xsd:float) ^ swrlb:greaterThan(?g,"3.9"^^xsd:float)-> hasWarningCode(?a, "G02") | Clinical expert experience |
| D703 | bg level 1 | PatientProfile(?p) ^ hasDiabetesDiagnosis(?p, true) ^ hasManageTask(?p,?m)^ hasAbnormalWarning(?m, ?a) ^ hasGlu(?p, ?g) ^ swrlb:lessThanOrEqual(?g, "22.2"^^xsd:float) ^ swrlb:greaterThan(?g,"16.7"^^xsd:float) | Clinical expert experience |
| D704 | DKA abnormal | PatientProfile(?p) ^ hasDiabetesDiagnosis(?p, true) ^ hasManageTask(?p,?m)^ hasAbnormalWarning(?m, ?a) ^ hasGlu(?p, ?g) ^ swrlb:lessThanOrEqual(?g, "22.2"^^xsd:float) ^ swrlb:greaterThan(?g,"16.7"^^xsd:float)^hasBloodKetone(?p,?b) | Clinical expert experience |
| D705 | bg level 2 | PatientProfile(?p) ^ hasDiabetesDiagnosis(?p, true) ^ hasManageTask(?p,?m)^ hasAbnormalWarning(?m, ?a)^ hasGlu(?p, ?g) ^ swrlb:greaterThan(?g, "22.2"^^xsd:float) -> hasWarningCode(?a, "G05") | Clinical expert experience |
| D706 | intolerance | PatientProfile(?p) ^ hasDiabetesDiagnosis(?p, true) ^hasManageTask(?p,?m)^ hasAbnormalWarning(?m, ?a) ^ hasDiscomfort(?p, true) -> hasWarningCode(?a, "D01") | Clinical expert experience |
| D707 | high blood pressure | Patient(?p)^hasBP(?p,?bp)^hasBPLevel(?bp,Severe)-> hasWarningCode (?a, "B01") | Clinical expert experience |
| D708 | Abnormal weekly blood pressure-2 | Patient(?p)^hasDiabetesDiagnosis(?p,true) ^hasManageTask(?p,?m)^hasAbnormalWarning(?m,?a) ^hasWBP(?p,?wbp)^hasBPLevel(?wbp,Severe)-> | Clinical expert experience |
| D709 | Abnormal weekly blood pressure-2 | PatientProfile(?p) ^ hasDiabetesDiagnosis(?p, true) ^hasManageTask(?p,?m)^ hasAbnormalWarning(?m, ?a) ^ hasWBP(?p,?wbp)^hasBPLevel(?wbp,Moderate) -> hasWarningCode(?a, "B03") | Clinical expert experience |
| D710 | Abnormal heart rate-1 | PatientProfile(?p) ^ hasDiabetesDiagnosis(?p, true) ^hasManageTask(?p,?m)^ hasAbnormalWarning(?m, ?a) ^ hasHR(?p,?hr)^swrlb:lessThan(?hr,50) -> hasWarningCode(?a, "B04") | Clinical expert experience |
| D711 | Abnormal heart rate-2 | PatientProfile(?p)^hasDiabetesDiagnosis(?p, true) ^hasManageTask(?p,?m)^ hasAbnormalWarning(?m, ?a) ^ hasHR(?p,?hr)^ swrlb:greaterThan(?hr,100) -> hasWarningCode(?a, "B05") | Clinical expert experience |
| D712 | Hypotension-1 | Patient(?p)^hasDiabetesDiagnosis(?p,true)^hasManageTask(?p,?m)  ^hasAbnormalWarning(?m,?a)^hasSBP(?p,?sbp)^ swrlb:lessThan(?sbp,90)->hasWarningCode(?a , "B06") | Clinical expert experience |
| D713 | Hypotension-2 | Patient(?p)^hasDiabetesDiagnosis(?p,true) ^hasManageTask(?p,?m)^hasAbnormalWarning(?m,?a) ^hasDBP(?p,?dbp)^ swrlb:lessThan(?dbp,60)->  hasWarningCode(?a , "B07") | Clinical expert experience |
| D714 | Abnormal monthly blood pressure-1 | Patient(?p)^hasDiabetesDiagnosis(?p,true) ^hasManageTask(?p,?m)^hasAbnormalWarning(?m,?a) ^hasMBP(?p,?mbp)^hasBPLevel(?mbp,Severe)-> | Clinical expert experience |
| D715 | Abnormal monthly blood pressure-2 | PatientProfile(?p)^hasDiabetesDiagnosis(?p,true) ^hasManageTask(?p,?m)^hasAbnormalWarning(?m,?a)^ hasMBP(?p,?mbp)^hasBPLevel(?mbp,Moderate)-> hasWarningCode(?a, "B09") | Clinical expert experience |

Table 8 Expression results of SWRL rules for diabetes medication guidance

| serial | rules | rules definition | source |
| --- | --- | --- | --- |
| D801 | simple lifestyle intervention | PatientProfile(?p)^hasDiabetesDiagnosis(?p,true)^hasManageTask  (?p,?t)^hasLifestyleIntervention(?t,?l)^hasLifestyleSubplan(?l,?s)  ^hasLifestyleIntervention(?l) | Guidelines for the prevention and control of type 2 diabetes in China  Page:304 key points |
| D802 | single drug regimen | Patient(?p)^needMedication(?p,true)^hasAvgGlu(?p,?glu)^hasGluLevel(? glu,Mild)->medicationStatus(?p,1) | Guidelines for the prevention and control of type 2 diabetes in China  Page:304 key points |
| D803 | double drug dosage-1 | Patient(?p)^needMedication(?p,true)^hasAvgGlu(?p,?glu)^hasGluLevel(? glu,Moderate)->medicationStatus(?p,2) | Guidelines for the prevention and control of type 2 diabetes in China  Page:304 key points |
| D804 | double drug dosage-2 | Patient(?p)^needMedication(?p,true)^hasAvgGlu(?p,?glu)^hasGluLevel(?glu,Severe)->medicationStatus(?p,2) | National guidelines for the prevention and control of diabetes in primary care  Page:889 paragraph: 4.4 |
| D805 | Triple drug dosage | Patient(?p)^medicationStatus(?p,2)^hasTakenMedicine(?p,30)^hasAvgGlu(?p,?glu)^Dysarteriotony(?glu) ->medicationStatus(?p,3) | Guidelines for the prevention and control of type 2 diabetes in China  Page:304 key points |
| D806 | insulin | Patient(?p)^medicationStatus(?p,3)^hasTakenMedicine(?p,30)^hasAvgGlu(?p,?glu)^Dysarteriotony(?glu) ->medicationStatus(?p,4) | National guidelines for the prevention and control of diabetes in primary care  Page:889 paragraph: 4.3.5 |
| D807 | Insulin+ Hypoglycemic drug | Patient(?p)^medicationStatus(?p,4)^hasTakenMedicine(?p,30)^hasAvgGlu(?p,?glu)^Dysarteriotony(?glu) ->medicationStatus(?p,5) | National guidelines for the prevention and control of diabetes in primary care  Page:889 paragraph: 4.4 |
| D808 | Metformin | Patient(?p)^hasMedicine(?p,?m)^swrlb:equal(?m,metformin)^hasDose(?m,?d)^swrlb:greaterThan(?d,500)^swrlb:lessThan(?d,2000)->overDose(?p,?m) | Guidelines for the prevention and control of type 2 diabetes in China  Page:304 paragraph: 11.1.1 |
| D809 | Metformin Hydrochloride Sustained Release Tablets | Patient(?p)^hasMedicine(?p,?m)^swrlb:equal(?m,metformintablets)^hasDose(?m,?d)^swrlb:greaterThan(?d,500)^swrlb:lessThan(?d,2000)->overDose(?p,?m) | Guidelines for the prevention and control of type 2 diabetes in China  Page:304 paragraph: 11.1.1 |
| D810 | Glibenclamide | Patient(?p)^hasMedicine(?p,?m)^swrlb:equal(?m,sulfonylureas)^hasDose(?m,?d)^swrlb:greaterThan(?d,2.5)^swrlb:lessThan(?d,20)->overDose(?p,?m) | Guidelines for the prevention and control of type 2 diabetes in China  Page:342 appendix: 5 |
| D811 | Glibenclamide | Patient(?p)^hasMedicine(?p,?m)^swrlb:equal(?m,sulfonylureas)^hasDose(?m,?d)^swrlb:greaterThan(?d,2.5)^swrlb:lessThan(?d,20)->overDose(?p,?m) | Guidelines for the prevention and control of type 2 diabetes in China  Page:342 appendix: 5 |
| D812 | gliclazide | Patient(?p)^hasMedicine(?p,?m)^swrlb:equal(?m,sulfonylureas)^hasDose(?m,?d)^swrlb:greaterThan(?d,80)^swrlb:lessThan(?d,320)->overDose(?p,?m) | Guidelines for the prevention and control of type 2 diabetes in China  Page:342 appendix: 5 |
| D813 | gliclazide Sustained Release Tablets | Patient(?p)^hasMedicine(?p,?m)^swrlb:equal(?m,sulfonylureas)^hasDose(?m,?d)^swrlb:greaterThan(?d,30)^swrlb:lessThan(?d,120)->overDose(?p,?m) | Guidelines for the prevention and control of type 2 diabetes in China  Page:342 appendix: 5 |
| D814 | Glipizide | Patient(?p)^hasMedicine(?p,?m)^swrlb:equal(?m,sulfonylureas)^hasDose(?m,?d)^swrlb:greaterThan(?d,2.5)^swrlb:lessThan(?d,30)->overDose(?p,?m) | Guidelines for the prevention and control of type 2 diabetes in China  Page:342 appendix: 5 |
| D815 | Glipizide Sustained Release Tablets | Patient(?p)^hasMedicine(?p,?m)^swrlb:equal(?m,sulfonylureas)^hasDose(?m,?d)^swrlb:greaterThan(?d,5)^swrlb:lessThan(?d,20)->overDose(?p,?m) | Guidelines for the prevention and control of type 2 diabetes in China  Page:342 appendix: 5 |
| D816 | Gliquidone | Patient(?p)^hasMedicine(?p,?m)^swrlb:equal(?m,sulfonylureas)^hasDose(?m,?d)^swrlb:greaterThan(?d,30)^swrlb:lessThan(?d,180)->overDose(?p,?m) | Guidelines for the prevention and control of type 2 diabetes in China  Page:342 appendix: 5 |
| D817 | glimepiride | Patient(?p)^hasMedicine(?p,?m)^swrlb:equal(?m,sulfonylureas)^hasDose(?m,?d)^swrlb:greaterThan(?d,1)^swrlb:lessThan(?d,8)->overDose(?p,?m) | Guidelines for the prevention and control of type 2 diabetes in China  Page:306 paragraph: 11.1.6 |
| D818 | Repaglinide | Patient(?p)^hasMedicine(?p,?m)^swrlb:equal(?m,grenadiers)^hasDose(?m,?d)^swrlb:greaterThan(?d,1)^swrlb:lessThan(?d,16)->overDose(?p,?m) | Guidelines for the prevention and control of type 2 diabetes in China  Page:305 paragraph: 11.1.4 |
| D819 | Nateglinide | Patient(?p)^hasMedicine(?p,?m)^swrlb:equal(?m,grenadiers)^hasDose(?m,?d)^swrlb:greaterThan(?d,120)^swrlb:lessThan(?d,360)->overDose(?p,?m) | Guidelines for the prevention and control of type 2 diabetes in China  Page:305 paragraph: 11.1.4 |
| D820 | Mitiglinide Calcium Tablets | Patient(?p)^hasMedicine(?p,?m)^swrlb:equal(?m,grenadiers)^hasDose(?m,?d)^swrlb:greaterThan(?d,30)^swrlb:lessThan(?d,60)->overDose(?p,?m) | Guidelines for the prevention and control of type 2 diabetes in China  Page:305 paragraph: 11.1.4 |
| D821 | acarbose | Patient(?p)^hasMedicine(?p,?m)^swrlb:equal(?m,α-glycosidase)^hasDose(?m,?d)^swrlb:greaterThan(?d,100)^swrlb:lessThan(?d,300)->overDose(?p,?m) | Guidelines for the prevention and control of type 2 diabetes in China  Page:342 appendix: 5 |
| D822 | Voglibose | Patient(?p)^hasMedicine(?p,?m)^swrlb:equal(?m,α-glycosidase)^hasDose(?m,?d)^swrlb:greaterThan(?d,0.2)^swrlb:lessThan(?d,0.9)->overDose(?p,?m) | Guidelines for the prevention and control of type 2 diabetes in China  Page:342 appendix: 5 |
| D823 | Miglitol | Patient(?p)^hasMedicine(?p,?m)^swrlb:equal(?m,α-glycosidase)^hasDose(?m,?d)^swrlb:greaterThan(?d,100)^swrlb:lessThan(?d,300)->overDose(?p,?m) | Guidelines for the prevention and control of type 2 diabetes in China  Page:342 appendix: 5 |
| D824 | Rosiglitazone Hydrochloride | Patient(?p)^hasMedicine(?p,?m)^swrlb:equal(?m,TZDs)^hasDose(?m,?d)^swrlb:greaterThan(?d,4)^swrlb:lessThan(?d,8)->overDose(?p,?m) | Guidelines for the prevention and control of type 2 diabetes in China  Page:305 paragraph: 11.1.3 |
| D825 | Pioglitazone | Patient(?p)^hasMedicine(?p,?m)^swrlb:equal(?m,TZDs)^hasDose(?m,?d)^swrlb:greaterThan(?d,15)^swrlb:lessThan(?d,45)->overDose(?p,?m) | Guidelines for the prevention and control of type 2 diabetes in China  Page:305 paragraph: 11.1.3 |
| D826 | Isophand or Protamine Zinc Insulin | Patient(?p)^hasMedicine(?p,?m)^swrlb:equal(?m,insulin)^hasDose(?m,?d)^swrlb:greaterThan(?d,0.1)^swrlb:lessThan(?d,0.3)->overDose(?p,?m) | Guidelines for the prevention and control of type 2 diabetes in China  Page:307 paragraph: 11.3.2 |
| D827 | pre-aerated Insulin | Patient(?p)^hasMedicine(?p,?m)^swrlb:equal(?m,insulin)^hasDose(?m,?d)^swrlb:greaterThan(?d,0.3)^swrlb:lessThan(?d,0.5)->overDose(?p,?m) | Guidelines for the prevention and control of type 2 diabetes in China  Page:307 paragraph: 11.3.3 |
| D828 | Enalapril | Patient(?p)^hasMedicine(?p,?m)^swrlb:equal(?m,enalapril)^hasDose(? m,?d)^swrlb:greaterThan(?d,20)->overDose(?p,?m) | Guidelines for the prevention and control of type 2 diabetes in China  Page:341 appendix: 4 |
| D829 | Captopril | Patient(?p)^hasMedicine(?p,?m)^swrlb:equal(?m,captopril)^hasDose(? m,?d)^swrlb:greaterThan(?d,50)->overDose(?p,?m) | Guidelines for the prevention and control of type 2 diabetes in China  Page:341 appendix: 4 |
| D830 | benazepril | Patient(?p)^hasMedicine(?p,?m)^swrlb:equal(?m,benazepril)^hasDose( ?m,?d)^swrlb:greaterThan(?d,20)->overDose(?p,?m) | Guidelines for the prevention and control of type 2 diabetes in China  Page:341 appendix: 4 |
| D831 | irbesartan | Patient(?p)^hasMedicine(?p,?m)^swrlb:equal(?m,irbesartan)^hasDose(? m,?d)^swrlb:greaterThan(?d,300)->overDose(?p,?m) | Guidelines for the prevention and control of type 2 diabetes in China  Page:341 appendix: 4 |
| D832 | telmisartan | Patient(?p)^hasMedicine(?p,?m)^swrlb:equal(?m,telmisartan)^hasDose( ?m,?d)^swrlb:greaterThan(?d,80)->overDose(?p,?m) | Guidelines for the prevention and control of type 2 diabetes in China  Page:341 appendix: 4 |
| D833 | Atenolol | Patient(?p)^hasMedicine(?p,?m)^swrlb:equal(?m,atenolol)->overDose(? p,?m) | Guidelines for the prevention and control of type 2 diabetes in China  Page:341 appendix: 4 |
| D834 | Metoprolol | Patient(?p)^hasMedicine(?p,?m)^swrlb:equal(?m,metoprolol)^hasDose( ?m,?d)^swrlb:greaterThan(?d,25)->overDose(?p,?m) | Guidelines for the prevention and control of type 2 diabetes in China  Page:341 appendix: 4 |
| D835 | amlodipine | Patient(?p)^hasMedicine(?p,?m)^swrlb:equal(?m,amlodipine) ^hasDose(?m,?d)^swrlb:greaterThan(?d,10)->overDose(?p,?m) | Guidelines for the prevention and control of type 2 diabetes in China  Page:341 appendix: 4 |
| D836 | Nifedipine | Patient(?p)^hasMedicine(?p,?m)^swrlb:equal(?m,nifedipine)^hasDose(? m,?d)^swrlb:greaterThan(?d,20)->overDose(?p,?m) | Guidelines for the prevention and control of type 2 diabetes in China  Page:341 appendix: 4 |
| D837 | Lacidipine | Patient(?p)^hasMedicine(?p,?m)^swrlb:equal(?m,lacidipine)^hasDose(? m,?d)^swrlb:greaterThan(?d,8)->overDose(?p,?m) | Guidelines for the prevention and control of type 2 diabetes in China  Page:341 appendix: 4 |
| D838 | Cilnidipine | Patient(?p)^hasMedicine(?p,?m)^swrlb:equal(?m,cilnidipine)^hasDose( ?m,?d)^swrlb:greaterThan(?d,10)->overDose(?p,?m) | Guidelines for the prevention and control of type 2 diabetes in China  Page:341 appendix: 4 |
| D839 | hydrochlorothiazide | Patient(?p)^hasMedicine(?p,?m)^swrlb:equal(?m,hydrochlorothiazide) ^hasDose(?m,?d)^swrlb:greaterThan(?d,25)->overDose(?p,?m) | Guidelines for the prevention and control of type 2 diabetes in China  Page:341 appendix: 4 |
| D840 | Atorvastatin | Patient(?p)^hasMedicine(?p,?m)^swrlb:equal(?m,statin) ^hasDose(?m,?d)^swrlb:greaterThan(?d,10)->overDose(?p,?m) | Guidelines for the prevention and control of type 2 diabetes in China  Page:343 appendix: 8 |
| D841 | fluvastatin | Patient(?p)^hasMedicine(?p,?m)^swrlb:equal(?m,statin) ^hasDose(?m,?d)^swrlb:greaterThan(?d,80)->overDose(?p,?m) | Guidelines for the prevention and control of type 2 diabetes in China  Page:343 appendix: 8 |
| D842 | pitavastatin | Patient(?p)^hasMedicine(?p,?m)^swrlb:equal(?m,statin) ^hasDose(?m,?d)^swrlb:greaterThan(?d,2)->overDose(?p,?m) | Guidelines for the prevention and control of type 2 diabetes in China  Page:343 appendix: 8 |
| D843 | Pravastatin | Patient(?p)^hasMedicine(?p,?m)^swrlb:equal(?m,statin) ^hasDose(?m,?d)^swrlb:greaterThan(?d,40)->overDose(?p,?m) | Guidelines for the prevention and control of type 2 diabetes in China  Page:343 appendix: 8 |
| D844 | fenofibrate | Patient(?p)^hasMedicine(?p,?m)^swrlb:equal(?m,betts) ^hasDose(?m,?d)^swrlb:greaterThan(?d,0.3)->overDose(?p,?m) | Guidelines for the prevention and control of type 2 diabetes in China  Page:343 appendix: 8 |
| D845 | Bezafibrate | Patient(?p)^hasMedicine(?p,?m)^swrlb:equal(?m,betts) ^hasDose(?m,?d)^swrlb:greaterThan(?d,0.6)->overDose(?p,?m) | Guidelines for the prevention and control of type 2 diabetes in China  Page:343 appendix: 8 |

Table 9 Expression results of SWRL rules for diabetes lifestyle guidance

| serial | rules | rules definition | source |
| --- | --- | --- | --- |
| D901 | sports | PatientProfile(?p)^hasDiabetesDiagnosis(?p,true)^hasManageTask(?p,?t)^hasLifestyleIntervention(?t,?l)^hasLifestyleSubplan(?l,?s)  ^hasExercise(?s) | Guidelines for the prevention and control of type 2 diabetes in China  Page:303 paragraph: 9 |
| D902 | diet | PatientProfile(?p)^hasDiabetesDiagnosis(?p,true)^hasManageTask(?p,?t)^hasLifestyleIntervention(?t,?l)^hasLifestyleSubplan(?l,?s)  ^hasDiet(?s) | Guidelines for the prevention and control of type 2 diabetes in China  Page:302 paragraph: 8 |
| D903 | Leisure sport | PatienteProfile(?p)^hasManageTask(?p.?t)^hasLifestyleIntervention(?t,?l)^hasLifestyleSubplan(?l,?s)^hasExercise (?s,?e)^hasRelax(?e,relaxSport)^swrlb:divide(?relaxSport,"0.035"^  ^xsd:double)->hasRelaxSportTime(?p,?sport) | Guidelines for the prevention and control of type 2 diabetes in China  Page:303 paragraph: 9 |
| D904 | Mild sports | PatienteProfile(?p)^hasManageTask(?p.?t)^hasLifestyleIntervention(?t,?l)^hasLifestyleSubplan(?l,?s)^hasExercise (?s,?e)^hasMild(?e,mildSport)^swrlb:divide(?mildSport,"0.07"^  ^xsd:double)->hasMildSportTime(?p,?sport) | Guidelines for the prevention and control of type 2 diabetes in China  Page:303 paragraph: 9 |
| D905 | Moderate sports | PatienteProfile(?p)^hasManageTask(?p.?t)^hasLifestyleIntervention(?t,?l)^hasLifestyleSubplan(?l,?s)^hasExercise(?s,?e)^hasModerate(?e, moderateSport)^swrlb:divide(?moderateSport,"0.035"^  ^xsd:double)->hasModerateSportTime(?p,?sport) | Guidelines for the prevention and control of type 2 diabetes in China  Page:303 paragraph: 9 |
| D906 | Severe sports | PatienteProfile(?p)^hasManageTask(?p.?t)^hasLifestyleIntervention(?t,?l)^hasLifestyleSubplan(?l,?s)^hasExercise (?s,?e)^hasServer(?e,serverSport)^swrlb:divide(?serverSport,"0.035"^  ^xsd:double)->hasServerSportTime(?p,?sport) | Guidelines for the prevention and control of type 2 diabetes in China  Page:303 paragraph: 9 |
| D910 | total calorie intake | PatientProfile(?p)^hasDiabetesDiagnosis(?p,true)^hasManageTask(?p,?t)^hasLifestyleIntervention(?t,?l)^hasLifestyleSubplan(?l,?s)  ^hasDiet(?s)^hasHeight(?p,?h)^swrlb:minus(?h, "105"^^xsd:double)->hasIdealWeight(?p,?iw)^swrlb:multiply(?iw, "25"^^xsd:double)->hasCalories(?calories) | Guidelines for the prevention and control of type 2 diabetes in China  Page:302 paragraph: 8.2 |
| D911 | calorie intake for breakfast | PatientProfile(?p)^hasDiabetesDiagnosis(?p,true)^hasManageTask(?p,?t)^hasLifestyleIntervention(?t,?l)^hasLifestyleSubplan(?l,?s)  ^hasDiet(?s)^hasHeight(?p,?h)^swrlb:minus(?h, "105"^^xsd:double)->hasIdealWeight(?p,?iw)^swrlb:multiply(?iw, "25"^^xsd:double)->hasCalories(?s,?calories) ^hasBreakfast(?b)  ^swrlb:multiply(?calories, "0.3"^^xsd:double)->  hasBreakfastCalories(?b,breakfast) | Both Clinical expert experience and medical guidelines |
| D912 | calorie intake for lunch | PatientProfile(?p)^hasDiabetesDiagnosis(?p,true)^hasManageTask(?p,?t)^hasLifestyleIntervention(?t,?l)^hasLifestyleSubplan(?l,?s)  ^hasDiet(?s)^ hasCalories(?s,?calories)^hasLunch(?b)  ^swrlb:multiply(?calories, "0.3"^^xsd:double)->  hasLunchCalories(?b,lunch) | Both Clinical expert experience and medical guidelines |
| D913 | calorie intake for dinner | PatientProfile(?p)^hasDiabetesDiagnosis(?p,true)^hasManageTask(?p,?t)^hasLifestyleIntervention(?t,?l)^hasLifestyleSubplan(?l,?s)  ^hasDiet(?s)^ hasCalories(?s,?calories)^hasDinner(?b)  ^swrlb:multiply(?calories, "0.3"^^xsd:double)->  hasDinnerCalories(?b,dinner) | Both Clinical expert experience and medical guidelines |

Table 10 Expression results of SWRL rules for diabetes compliance management

| serial | rules | rules definition | source |
| --- | --- | --- | --- |
| D1001 | poor compliance-1 | PatientProfile(?p)^hasDiabetesDiagnosis(?p,true)^ hasManageTask(?p,?m) ^ hasComplianceManagement(?m, ?c) ^ hasManagementLevel(?p, ?l) ^ hasComplianceProportion(?c, ?cp) ^ swrlb:equal(?cp, "0"^^xsd:integer) -> hasComplianceLevel(?c, "5"^^xsd:integer) | Clinical expert experience |
| D1002 | poor compliance-2 | PatientProfile(?p)^hasDiabetesDiagnosis(?p,true)^ hasManageTask(?p,?m) ^ hasComplianceManagement(?m, ?c) ^ hasManagementLevel(?p, ?l) ^ hasComplianceProportion(?c, ?cp) ^swrlb:greaterThan(?cp,"0"^^xsd:integer)^ swrlb:lessThanOrEqual(?cp,"0.5"^^xsd:decimal)-> hasComplianceLevel(?c, "4"^^xsd:integer) | Clinical expert experience |
| D1003 | average compliance | PatientProfile(?p)^hasDiabetesDiagnosis(?p,true)^ hasManageTask(?p,?m) ^ hasComplianceManagement(?m, ?c) ^ hasManagementLevel(?p, ?l) ^ hasComplianceProportion(?c, ?cp) ^swrlb:greaterThan(?cp,"0.5"^^xsd:decimal)^ swrlb:lessThanOrEqual(?cp,"0.8"^^xsd:decimal)-> hasComplianceLevel(?c, "3"^^xsd:integer) | Clinical expert experience |
| D1004 | Good compliance | PatientProfile(?p)^hasDiabetesDiagnosis(?p,true)^ hasManageTask(?p,?m) ^ hasComplianceManagement(?m, ?c) ^ hasManagementLevel(?p, ?l) ^ hasComplianceProportion(?c, ?cp) ^swrlb:greaterThan(?cp,"0.8"^^xsd:decimal)^ swrlb:lessThan(?cp,"1"^^xsd:decimal)-> hasComplianceLevel(?c, "2"^^xsd:integer) | Clinical expert experience |
| D1005 | excellent compliance | PatientProfile(?p)^hasDiabetesDiagnosis(?p,true)^ hasManageTask(?p,?m) ^ hasComplianceManagement(?m, ?c) ^ hasManagementLevel(?p, ?l) ^ hasComplianceProportion(?c, ?cp) ^ swrlb:equal(?cp, "1"^^xsd:integer) -> hasComplianceLevel(?c, "1"^^xsd:integer) | Clinical expert experience |
